# The probiotic *Lacticaseibacillus rhamnosus* GG supplementation reduces *Salmonella* load and modulates growth, intestinal morphology, gut microbiota, and immune responses in chickens

**DOI:** 10.1128/iai.00420-24

**Published:** 2025-04-02

**Authors:** Gary Closs, Menuka Bhandari, Yosra A. Helmy, Dipak Kathayat, Dhanashree Lokesh, Kwonil Jung, Isidora D. Suazo, Vishal Srivastava, Loic Deblais, Gireesh Rajashekara

**Affiliations:** 1 Center for Food Animal Health, Department of Animal Sciences, The Ohio State University 462953 , Columbus, Ohio, USA; 2 Department of Food Science & Technology, The Ohio State University 554455 , Columbus, Ohio, USA; University of Pennsylvania, Philadelphia, Pennsylvania, USA

**Keywords:** *Salmonella*, probiotics, LGG, chickens, foodborne pathogens

## Abstract

*Salmonella,* a leading cause of foodborne illnesses, is primarily transmitted to humans through the consumption of contaminated poultry products. The increasing resistance of *Salmonella* to antibiotics and lack of cross-protection by vaccines necessitate new control strategies in poultry production systems. This study assessed the efficacy of probiotics against *Salmonella* Typhimurium (ST) and *Salmonella* Enteritidis (SE). *Lactobacillus acidophilus* (LA), *Lacticaseibacillus rhamnosus* GG (LGG), and *Bifidobacterium animalis* subsp. lactis (Bb12) showed inhibition of ST and SE in agar well diffusion assay, with stable inhibitory properties. In co-culture assay, both LGG and Bb12 completely suppressed ST and SE growth. Liquid chromatography-with tandem mass spectrometry (LC-MS/MS) analysis of the LGG and Bb12 cell-free culture supernatant identified novel bioactive peptides with anti-*Salmonella* properties. Administering LGG in drinking water of chickens raised on built-up litter floor in experimental conditions significantly reduced the ST load (5.95 logs and 3.74 on 7 days post-infection [dpi] and 14 dpi, respectively). Gut microbiota analysis revealed increased abundance of several beneficial genera such as *Butyricicoccus*, *Erysipelatoclostridium*, *Flavonifractor,* and *Bacillus* in LGG-treated groups. Histomorphometry analysis demonstrated increased villus height (VH) and VH by crypt depth ratio in the ileum of the LGG-treated group on 14 dpi. These results highlight LGG as a promising probiotic for controlling *Salmonella* in chickens and reducing transmission to humans. The beneficial properties of LGG are attributed to the production of antimicrobial peptides, microbiota modulation, and enhanced intestinal integrity.

**IMPORTANCE:**

Salmonella is the leading cause of foodborne illnesses in the United States and worldwide. It is primarily transmitted through contaminated poultry and poultry products (eggs and poultry meat). Increasing resistance of Salmonella to antibiotics and lack of cross-protection by vaccines necessitate new control strategies to reduce Salmonella in poultry production system and minimize human infections. Probiotics, which are live beneficial microorganisms when administered in an optimum amount, have been increasingly used in recent years as alternatives to antibiotics to promote health. Our study showed that LGG exhibited superior probiotics properties and significantly reduced Salmonella load in chickens. Thus, LGG supplementation is a promising approach to prevent Salmonella infection and enhance performance of poultry thereby enhance food safety, proper antibiotic stewardship and public health.

## INTRODUCTION

*Salmonella* is a rod-shaped, Gram-negative, facultative anaerobic bacteria that causes salmonellosis. The genus is divided into two species: *Salmonella bongori* and *Salmonella enterica* (1). Non-typhoidal *Salmonella* (NTS) is the major cause of foodborne illnesses and gastroenteritis in humans. Globally, NTS gastroenteritis remains a concern with over 93 million cases and 155,000 deaths each year (2). According to the Centers for Disease Control and Prevention (CDC), *Salmonella* causes approximately 1.35 million infections, 26,500 hospitalizations, and 420 deaths each year in the United States (3). The total economic losses accounted by *Salmonella* were $4.1 billion in 2021 in the United States (4). *Salmonella* is classified into serotypes based on the surface antigens, with more than 2,600 serotypes of *Salmonella* recognized. Among them, *Salmonella enterica* serovar Typhimurium (ST) and *Salmonella enterica* serovar Enteritidis (SE) (non-typhoid serovars) are the most prevalent serotypes isolated from human infections; accounting for 16% and 20% of US human salmonellosis from 2004 to 2016, respectively (5–7).

According to the CDC, poultry and poultry products are the most common sources of *Salmonella* transmission in humans (8, 9). A study found that 19% and 15% of human salmonellosis cases were associated with poultry and eggs in the United States, respectively (10). In poultry, the pathogenicity of *Salmonella* depends on various factors such as strain and serotype, host, breed, and age; with higher chances of infection within the first 24 h of life (7). Birds can be infected with *Salmonella* vertically or horizontally. Vertical transmission of *Salmonella* occurs through eggs, whereas horizontal transmission occurs from the contaminated environment. Generally, birds infected with *Salmonella* do not show clinical symptoms; however, the bacteria can persist in the intestinal tract for extended periods (11, 12). The shedding of *Salmonella* by the infected birds leads to contamination of the environment. Direct or indirect contact with infected birds and contaminated environment can transmit *Salmonella* to humans. Numerous outbreaks of human salmonellosis have been reported from poultry (13). Thus, there is a necessity to control *Salmonella* infection in poultry to safeguard animal and human health, as well as to ensure the production of safe food.

The poultry production sector ranks as the second-highest consumer of antibiotics, following the pig production sector (14). Antibiotics belonging to fluoroquinolones are used to control *Salmonella* infection in poultry farms (15) and to promote growth and enhance production. The extensive use of fluoroquinolones and other antibiotics in food-animal production systems has resulted in the evolution and spread of multidrug-resistant *Salmonella* (16). A study conducted in Brazil found that *Salmonella* recovered from chickens carried 11 different anti-microbial resistant (AMR) genes (17). Furthermore, the same study reported the recovery of colistin resistance genes and extended spectrum beta-lactamases genes in *Salmonella* isolated from poultry and their products (17). Due to the concern of evolution of AMR to medically important antibiotics in farms and their potential transfer to humans, the Food and Drug Administration (FDA) banned the use of fluoroquinolones in poultry production (18). Additionally, the FDA banned the use of antibiotics for growth promotion (70 FR 44105; FDA GFI #209; FDA, 2013). Furthermore, administration of antibiotics can alter the beneficial microbes of the gut, disrupt the gut microbiome, and affect the overall health of poultry (19). While there is a need to limit antibiotic-resistant bacteria, the reduction in the use of antibiotics on the farm may increase the prevalence of foodborne pathogens and their transmission to humans (20). Currently, vaccines available to protect birds from salmonellosis do not provide cross-protection to heterologous serotypes of *Salmonella* (21). Moreover, biosecurity measures, which are effective up to a certain degree, are insufficient to completely eradicate *Salmonella*. Thus, there exists a critical need to find innovative and effective alternative antibacterial approaches to mitigate the problem of AMR *Salmonella* (22, 23).

Probiotics are live microorganisms that can offer health benefits to the host when administered in adequate amounts (24, 25). Probiotics are resistant to gastric acidity and bile salt environment, replicate in the gastrointestinal tract, and improve the intestinal integrity and gut microbiota composition (26). In poultry, probiotics are commonly used to improve animal growth, increase feed conversion efficiency, and reduce the pathogen load (27, 28). Notably, lactic acid bacteria (*Lactobacillus* and *Bifidobacterium*) are the major beneficial probiotics that possess anti-*Salmonella* activity (29, 30). Although *Bifidobacteria* and *Lactobacillus* have been positively correlated with beneficial host health (29, 31–33), the antimicrobial activity of a probiotic is strain-specific.

However, there is an overall lack of studies specifically demonstrating the activity of probiotic species against *Salmonella*. Furthermore, information is also lacking on the bioactive substances secreted/released by these probiotics as well as their interactions with commensal microbes and/or pathogens in the gut, which limits the understanding of the probiotic’s mechanism(s) of action and reproducible use in industrial settings. This study evaluated the efficacy of different probiotics (*Escherichia coli* Nissle 1917 [EcN], *Lacticaseibacillus rhamnosus* GG [LGG], *Lactobacillus acidophilus* [LA], *Levilactobacillus brevis* [Lbrev]*,* and *Bifidobacterium animalis* subsp. *lactis* [Bb12]) in inhibiting ST and SE under *in vitro* conditions. Based on the *in vitro* results, several antimicrobial peptides (AMPs) were isolated from the supernatant of the LGG and Bb12 (best-performing probiotics *in vitro*), and their inhibitory activity against *Salmonella* was characterized. Similarly, the *in vivo* efficacy of probiotics was assessed through oral administration (oral gavage and drinking water) to the *Salmonella*-infected chickens housed in cages as well as raised on built-up litter to mimic field-simulated conditions.

## RESULTS

### Whole culture of LGG, Bb12, and LA possessed anti-*Salmonella* property

A whole culture of probiotic bacteria was used in an agar well diffusion assay to screen for anti-*Salmonella* activity against ST and SE. Whole cultures of LGG and Bb12 were able to inhibit (14 mm and 14.5 mm inhibition zone, respectively) ST at 24 h (Table 1) (34). LA showed inhibition against ST comparable to LGG and Bb12 at 12 h but the zone of inhibition decreased to 11.5 mm by 24 h. Similar results were observed for SE with the inhibition zone of 11.5, 14.5, and 11.5 mm by LGG, Bb12, and LA, respectively, at 24 h. All tested probiotics, except EcN, showed inhibition against both ST and SE after 24 h (Table 1). Therefore, EcN was omitted from further analysis. At 12 h Lbrev demonstrated inhibition against both ST and SE, but no inhibition was observed at 24 h. Overall, the zone of inhibition observed at 24 h was smaller, which might be due to the absence of continuous production of inhibitory substances in solid media by probiotic bacteria, as stationary phase-grown cultures were used in the assay.

**TABLE 1:** Inhibition zones induced by whole culture (WC) of probiotics against ST and SE in an agar-well diffusion assay^*a*^

Salmonella	Probiotic bacteria candidates	Zone of inhibition (mm)					
		37°C WC		Effect of temperature			
				4°C WC		121°C WC	
		12 h	24 h	12 h	24 h	12 h	24 h
ST	LA	14	11.5	14	11.5	13	11.5
	L GG	15.5	14	15.5	14	12	10
	Bb12	16	14.5	16	14	13.5	11
	EcN^*b*^	0	0	–	–	–	–
	Lbrev	10	0	9.5	0	0	0
SE	LA	14	11.5	14	11.5	13	11.5
	LGG	15.5	11.5	15.15	11.5	12	10
	Bb12	16	14.5	16	14	13.5	11
	EcN^*b*^	–	–	–	–	–	–
	Lbrev	10	0	10	0	0	0

^*a*^ LA, *Lactobacillus acidophilus;* LGG, *Lacticaseibacillus rhamnosus* GG; Bb12, *Bifidobacterium animalis* subsp. lactis; EcN, *Escherichia coli* Nissle 1917; Lbrev, *Levilactobacillus brevis*.

^*b*^ –, not subjected to temperature treatments.

To assess the effect of thermal treatment on the anti-*Salmonella* property, probiotics were treated at 121°C and 4°C. Our results indicated that both heating and cooling the probiotics did not alter the anti-*Salmonella* properties of LA, LGG, and BB12 because the zone of inhibition remained similar before and after the thermal treatment (Table 1). In contrast, Lbrev treated at 121°C demonstrated no inhibition against ST and SE at 12 and 24 h. However, Lbrev treated at 4°C inhibited ST and SE at 12 h, but the inhibition was lost at 24 h.

### LA, LGG, and Bb12 significantly inhibited ST and SE in co-culture assays

**Fig 1:**
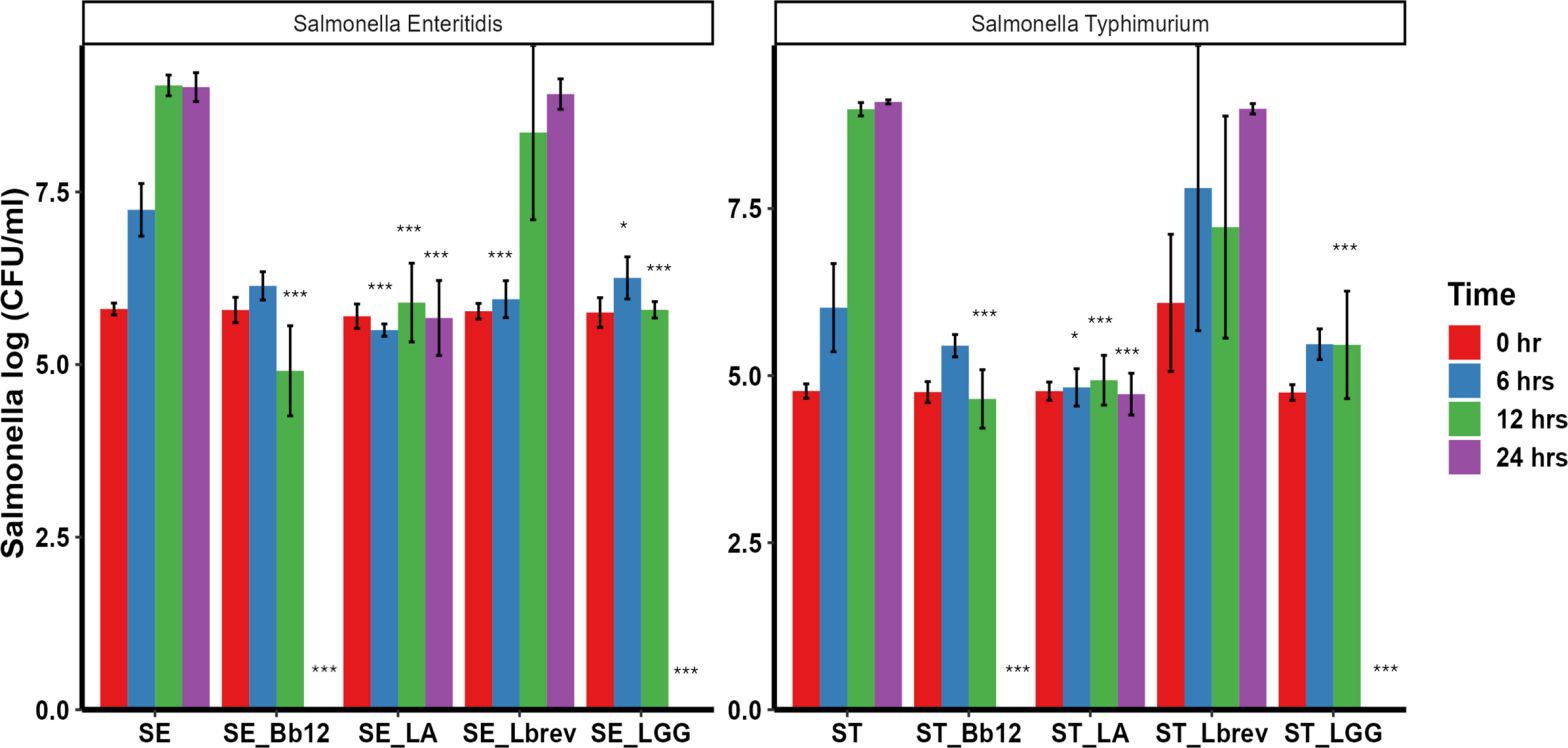
Growth and persistence of ST and SE in mixed media (mixture of MRS and LB at a ratio of 1:1) and co-cultured with LA, LGG, Bb12, and Lbrev. Each probiotic (100 µL of 10^8^ CFU/mL) was individually co-cultured with ST and SE (100 µL of ~5 × 10^7^ CFU/mL) and incubated anaerobically for 24 h at 37°C. ST and SE were enumerated at 0, 6, 12, and 24 h in co-culture assay.

Probiotics were co-cultured with a *Salmonella* strain to investigate their ability to inhibit the growth of ST and SE. The growth of *Salmonella* was not compromised in co-culture (100% MRS + 100% LB) media. In both the ST-LA and SE-LA co-cultures, LA began to significantly inhibit the growth of *Salmonella* as early as 6 h (Fig. 1). The co-culture of LA with ST significantly reduced ST by 1 log at 6 h (*P* < 0.05), while by 12 h the reduction of ST increased to 4 logs and remained the same throughout the study, *P* < 0.001 (Fig. 1). Co-culturing LA with SE significantly reduced SE at 6 h (1.7 log; *P* < 0.001). By 12 h the reduction of SE increased (3.15 logs; *P* < 0.001) and remained significantly lower (~3 logs; *P* < 0.001) than the SE alone at 24 h (Fig. 1). Although LGG and Bb12 showed no significant inhibition of ST at 6 h, by 12 h LGG significantly inhibited ST by 3.5 logs (*P* < 0.001) and SE by 3.25 logs (*P* < 0.001) (Fig. 1). Similarly, at 12 h, Bb12 inhibited ST and SE by 4 logs (*P* < 0.001) (Fig. 1). Notably, both LGG and Bb12 fully cleared ST and SE at 24 h (*P* < 0.001) (Fig. 1). Unlike other probiotics, Lbrev did not significantly reduce or clear ST at any of the measured time points (0, 6, 12, and 24 h) (Fig. 1). However, Lbrev significantly inhibited SE at 6 h, but not at any other measured time points (Fig. 1). Since LGG and Bb12 showed greater inhibition compared to LA at 24 h, LA was not included in further analysis (Fig. 1).

The pH of each co-culture became more acidic over time, suggesting that pH may play a role in *Salmonella* inhibition (Table 2). Although the pH of the Lbrev co-cultures reached an acidic environment (ST-Lbrev 4.79 ± 0.10; SE-Lbrev 4.88 ± 0.07) (Table 2), it did not inhibit ST or SE at 24 h. This is in direct contradiction to the co-cultures of LA, LGG, and Bb12 (ST-LA 4.57 ± 0.10; SE-LA 4.60 ± 0.17), (ST-LGG 4.29 ± 0.13; SE-LGG 4.36 ± 0.10), (ST-Bb12 4.20 ± 0.03; SE-Bb12 4.30 ± 0.09), which all inhibited ST and SE; implying pH is not the only factor involved in inhibition and suggesting the need to further evaluate the secreted products for antibacterial activity.

**TABLE 2:** pH of the mono and co-culture media over time^*a*^

Strains	Average pH			
	0 h	6 h	12 h	24 h
ST-LA	6.84 ± 0.06	6.18 ± 0.09	4.92 ± 0.15	4.57 ± 0.10
ST-LGG	6.81 ± 0.10	5.91 ± 0.30	4.65 ± 0.16	4.29 ± 0.13
ST-BB12	6.85 ± 0.06	5.69 ± 0.31	4.50 ± 0.13	4.20 ± 0.03
ST-Lbrev	6.85 ± 0.03	5.53 ± 0.13	4.98 ± 0.11	4.79 ± 0.10
ST	6.90 ± 0.04	6.70 ± 0.27	5.48 ± 0.33	4.91 ± 0.17
SE-LA	6.70 ± 0.28	5.96 ± 0.11	4.94 ± 0.29	4.60 ± 0.17
SE-LGG	6.72 ± 0.26	5.23 ± 0.15	4.40 ± 0.00	4.36 ± 0.10
SE-BB12	6.69 ± 0.27	5.15 ± 0.26	4.36 ± 0.08	4.30 ± 0.09
SE-Lbrev	6.76 ± 0.16	5.26 ± 0.13	5.06 ± 0.05	4.88 ± 0.07
SE	6.75 ± 0.27	6.61 ± 0.41	5.91 ± 0.29	5.42 ± 0.04

^*a*^ Measurements in pH ± SD. ST, *Salmonella* Typhimurium; SE, *Salmonella* Enteritidis; LA, *Lactobacillus acidophilus;* LGG, *Lacticaseibacillus rhamnosus* GG; Bb12, *Bifidobacterium animalis* subsp. *lactis*; EcN, *Escherichia coli* Nissle 1917; Lbrev, *Levilactobacillus brevis*.

### Secreted products of LGG and Bb12 possessed inhibitory characteristics in a trans-well assay

**Fig 2:**
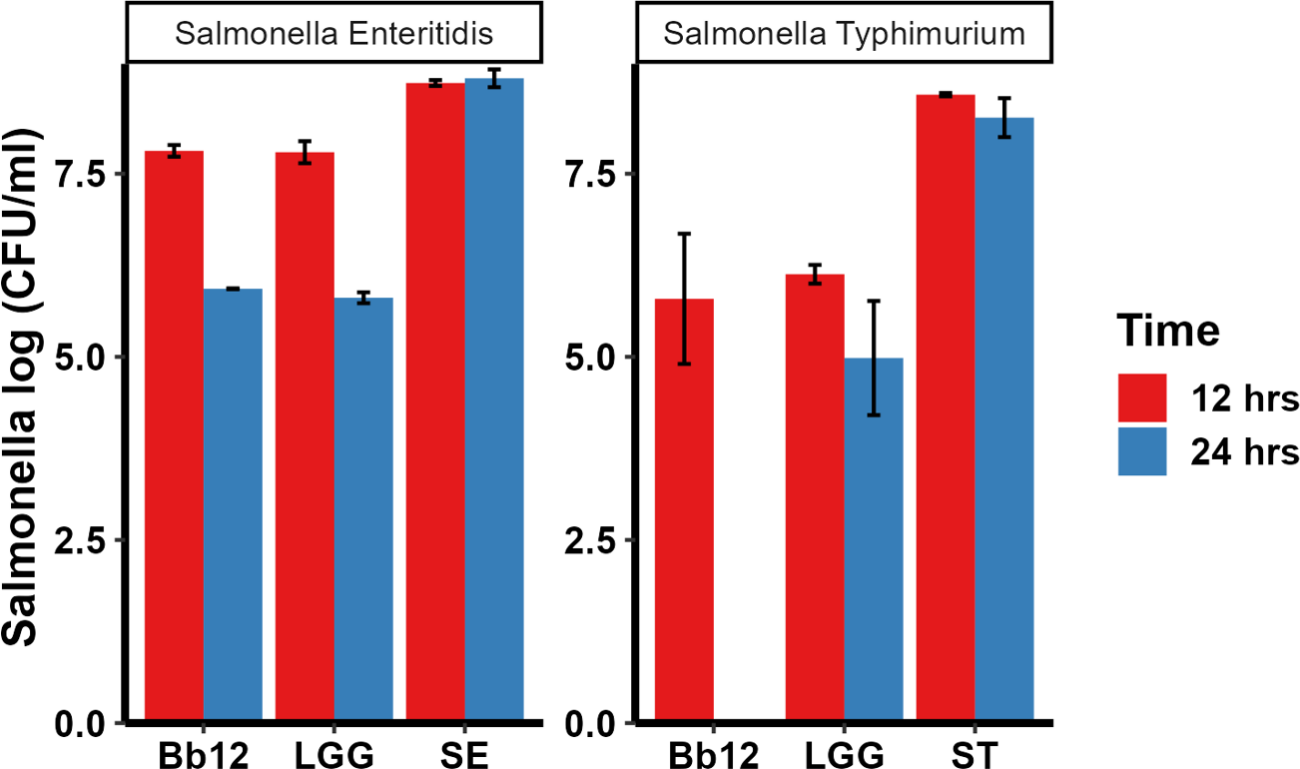
Inhibition of ST and SE by probiotics secreted products in a trans-well migration assay. *Salmonella* was added into a microcentrifuge tube containing 0.22 µm filter, and LGG and BB12 were added above the filter. *Salmonella* grown below and above the filter was used as control.

The secreted products of LGG and BB12 were tested in a trans-well assay to determine if the antagonistic effects are due to the bacteria secreted or released products. The secreted products of LGG and Bb12 yielded a 2.45 and 2.79 log reduction of ST at 12 h compared to the ST control (Fig. 2) in a trans-well assay. At 24 h, the effect was higher with ~3.3 logs reduction for LGG, while no viable ST were detected with Bb12 (Fig. 2). Although no secreted products of any probiotics caused a complete inhibition of SE there was still a noticeable difference. At 24 h both LGG and Bb12 caused an ~3 log reduction of SE (Fig. 2). These results suggested that the secreted products of the probiotics have anti-*Salmonella* properties.

### Antimicrobial properties of secreted products of LGG and BB12 were heat and protease resistant

The secreted or released products were further analyzed in an agar well diffusion assay to inhibit *Salmonella*. The cell-free supernatant (CFS) of LGG and Bb12 both resulted in a zone of inhibition against ST and SE of 12.5 mm at 12 h (Table 3). At 24 h, LGG and Bb12 yielded 9 mm (Table 3) against both ST and SE. There was not much difference in the zone of inhibition at 24 h when the CFS was heated to 121°C or cooled to 4°C, suggesting the temperature tolerance of the secreted products. The CFS of LGG and Bb12 was passed through a 3 kDa Amicon Ultra centrifugal filter, and the fractionated product still inhibited ST in an agar well diffusion assay (Table 3). When treated with proteinase K, the secreted products of LGG and Bb12 retained anti-*Salmonella* (ST inhibition: LGG and BB12 9 mm and SE inhibition: LGG and Bb12 9.5 mm) (Table 3) abilities at 24 h, suggesting that secreted or released antimicrobial products are heat stable, proteolysis resistant, and of low molecular weight in size.

**TABLE 3:** Inhibition zones induced by temperature and proteinase-K treated CFS against ST and SE in agar-well diffusion assay^*a*^

Probiotic bacteria candidates (CFS)	4°C CFS		CFS		121°C CFS		Proteinase K		<3 kDa filtrate	
	12 h	24 h	12 h	24 h	12 h	24 h	12 h	24 h	12 h	24 h
*Salmonella typhimurium* (ST)										
LGG	12.5	9	12.5	9	13	9	12	9	12.5	9
BB12	12	9.5	12.5	9	12.5	9.5	11	9	12.5	9
*Salmonella Enteritidis* (SE)										
LGG	12.5	9	12.5	9	13	9	12	9	ND^*b*^	ND^*b*^
BB12	12	9.5	12.5	9	12.5	9.5	11	9	ND^*b*^	ND^*b*^

^*a*^ ST, *Salmonella* Typhimurium; SE, *Salmonella* Enteritidis; LGG, *Lacticaseibacillus rhamnosus* GG; Bb12, *Bifidobacterium animalis* subsp. *lactis*.

^*b*^ ND, not determined.

### Cell-free supernatant of probiotics reduced invasion of *Salmonella* in polarized HT-29 cells

**Fig 3:**
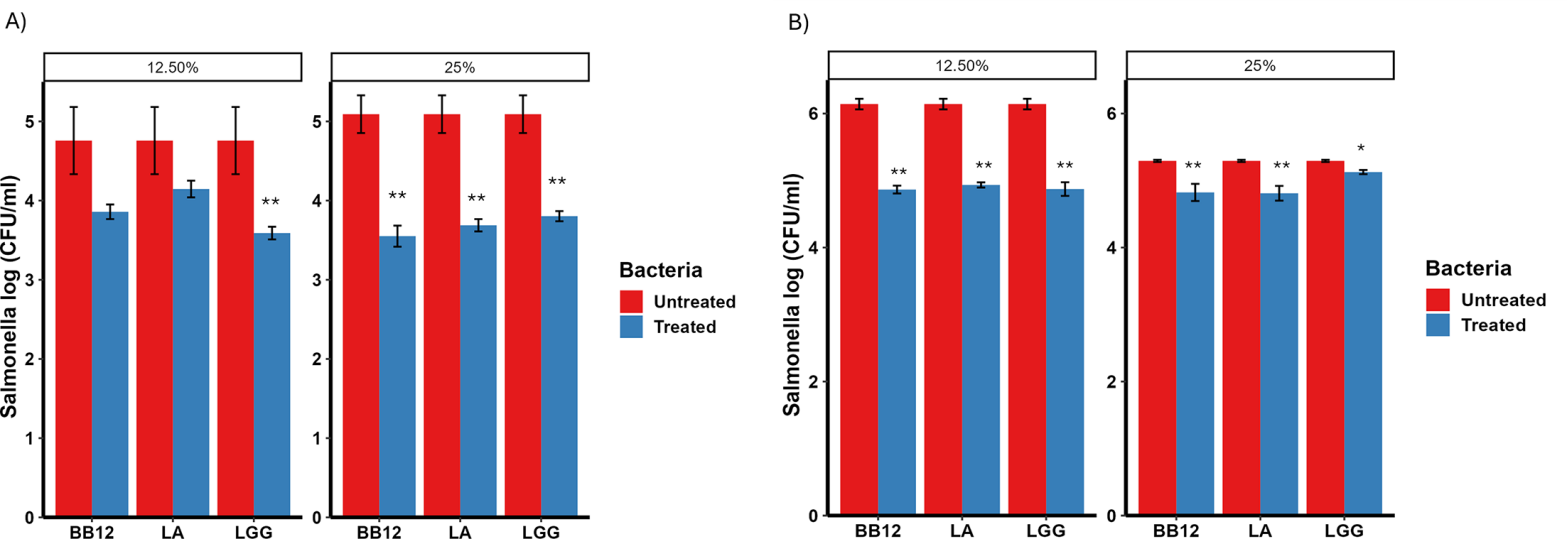
Effect of probiotic CFS to minimize invasion of ST (A) and SE (B) in HT-29 cells. Polarized HT-29 cells were infected with *Salmonella enterica* subsp. *enterica* serotype Typhimurium (A) and *Salmonella enterica* subsp. *enterica* serotype Enteritidis (B) and treated for 4 h with 12.5% and 25% CFS to determine the effect of probiotics on the invasion of *Salmonella*.

To further analyze the efficacy of the CFS of probiotics, CFS of LA, LGG, and BB12 were analyzed in a cell culture model. LA was included in the invasion assay because the whole culture of LA also showed inhibition of *Salmonella* in the agar-well diffusion assay (Table 1). Polarized HT-29 cells infected with *Salmonella* were treated with 12.5% and 25% CFS of LA, LGG, and Bb12. The 12.5% and 25% CFS of the probiotics were not toxic to the cells and non-inhibitory to *Salmonella* growth (data not shown). In the ST infected cells, 25% of CFS was most effective against *Salmonella* (Fig. 3A), while 12.5% of the CFS yielded the larger reduction in SE infected cells (Fig. 3B). There was a 1.29, 1.4, and 1.5 log reduction of ST from 25% CFS of LA, LGG, and Bb12, respectively (*P* < 0.05) (Fig. 3A). There was a 1.3, 1.27, and 1.28 log reduction of SE from 12.5% CFS of LA, LGG, and Bb12, respectively (*P* < 0.05) (Fig. 3B). However, no effect on the invasion was observed when HT-29 cells were pre-treated with LGG and Bb12 cells themselves after CFSs were separated and cells were resuspended in Dulbecco’s modified Eagle’s medium (DMEM) (data not shown).

### Oral administration of LGG reduced colonization of ST in the cecum of chickens (chicken trial 1)

**Fig 4:**
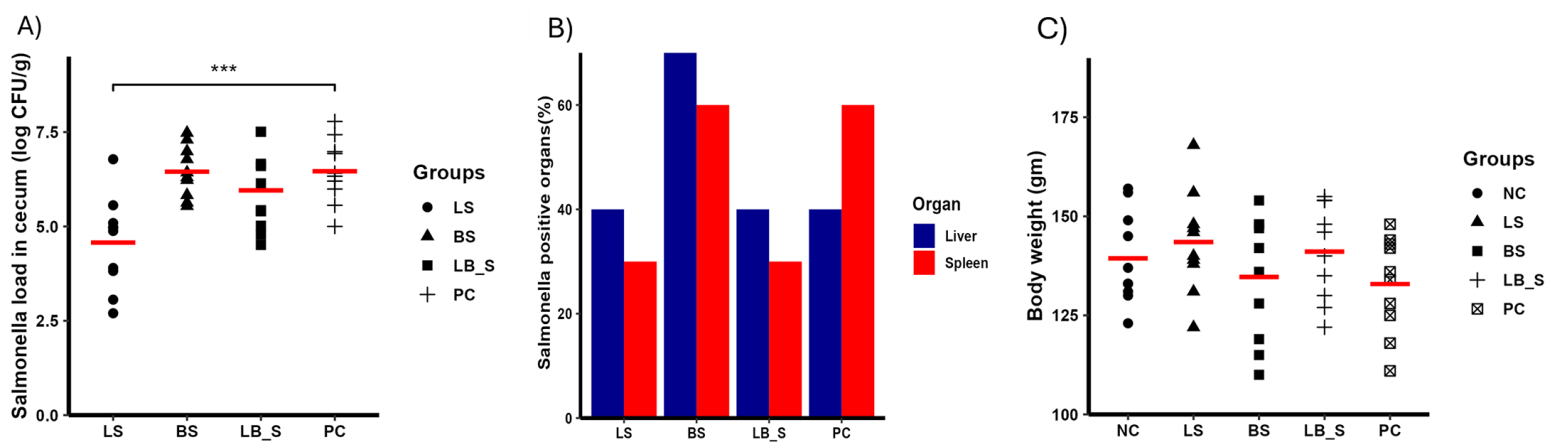
Impact of oral administration of probiotic against ST colonization in chicken. Birds were treated with LGG for 16 days and orally challenged with 10^4^ CFU nalidixic acid-resistant (Nal^r^) ST on day 7. The five groups included were NC (neither infected nor treated), LS (infected with *Salmonella* and treated with LGG), BS (infected with *Salmonella* and treated with Bb12), LB_S (infected with *Salmonell*a and treated with a mixture of LGG and Bb12 at the ratio of 1:1), and PC (infected with *Salmonella*). (A) Log CFU of ST colonization in cecum, (B) percent positive for ST in spleen and liver, and (C) body weight 10 days post-infection. *N* = 10/group; horizontal line: mean.

In this pilot study, we evaluated the effects of LGG and Bb12 on ST-infected chickens. The probiotics (LGG, Bb12, and LGG+Bb12 (1:1 combination)) were administered orally for 16 days (10^8^ CFU/chicken daily). The groups were designated as follows: NC (not treated and not challenged with ST), LS (treated with LGG and infected with *Salmonella*), BS (treated with Bb12 and infected with *Salmonella*), LB_S (treated with a mixture of LGG and Bb12 and infected with *Salmonella*), and PC (not treated and ST challenged). LGG (LS group) reduced *Salmonella* in the cecum by ~1.9 logs (*P* < 0.001) (Fig. 4A) on 10 days post-infection (dpi). The LS group also showed a 30% reduction in the number of birds positive for ST in spleen compared to the PC group (Fig. 4B). Neither the BS nor the LB_S groups were able to reduce the load of ST in the cecum. However, the LB_S treatment reduced the percent of birds positive for ST in spleen by 30% (Fig. 4B). None of the treatment groups reduced ST in the liver (Fig. 4B). Probiotic treatment did not alter body weights of birds (Fig. 4C).

### Supplementation of LGG in the drinking water reduced *Salmonella* load in the cecum of chickens raised in cages (chicken trial 2)

**Fig 5:**
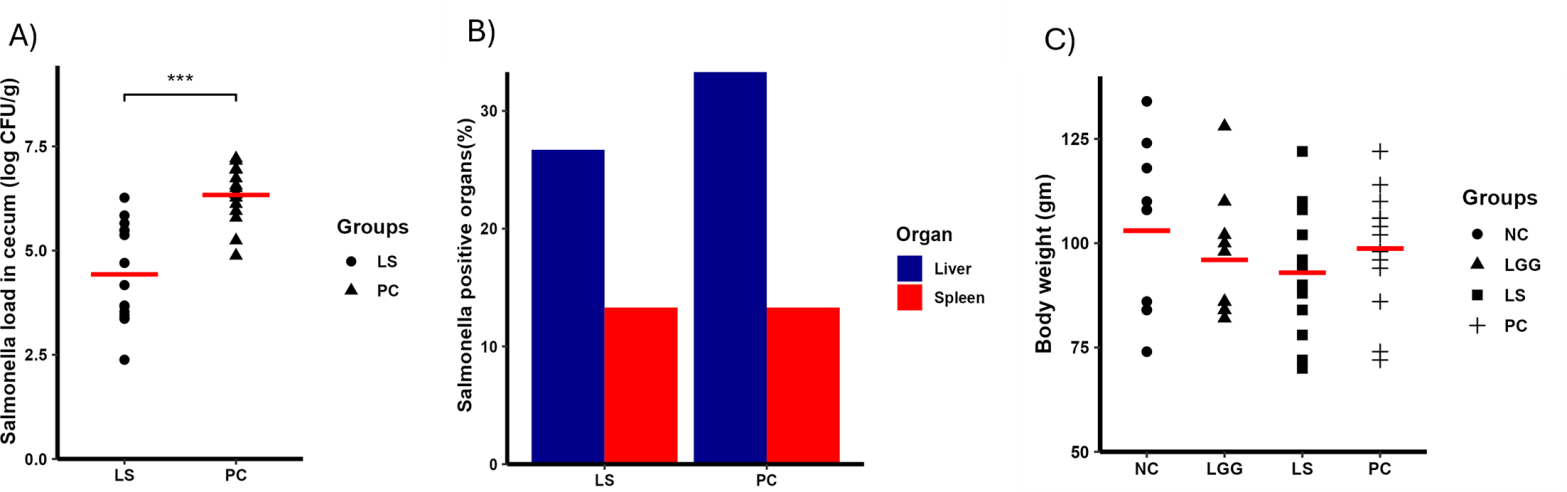
Efficacy of LGG supplemented in drinking water against ST colonization in chickens. Birds were treated with LGG for 13 days and orally challenged with 10^4^ CFU Nal^r^ ST on day 7. The four groups included were NC, LGG (not infected but treated with LGG), LS, and PC. (A) Log CFU of ST colonization in cecum, (B) percent positive for ST in spleen and liver, and (C) body weight 8 days post-infection, *N* = 15/group.

LGG was supplemented continuously in the drinking water of one-day-old specific pathogen-free (SPF) layers for 13 days to further optimize the delivery of probiotics to chickens. The different groups were NC, LGG (treated with LGG but not infected with *Salmonella*), LS, and PC. Similar to the above pilot chicken trial, LGG treated (LS group) reduced ST by ~1.91 logs in the cecum 8 dpi compared to untreated PC (*P* < 0.001) (Fig. 5A). The colonization of ST in spleen and liver was inconsistent (Fig. 5B). In the spleen, 13.3% of both LS and PC birds were positive for ST (Fig. 5B). In the liver, 26.7% of LS-treated birds and 33.3% of PC birds were positive for ST (Fig. 5B). Consistent with the previous trial, LGG treatment did not affect the body weight of the birds (Fig. 5C).

### Supplementation of LGG in drinking water of chickens raised on the floor reduced the *Salmonella* in cecum, liver, and spleen (chicken trial 3)

**Fig 6:**
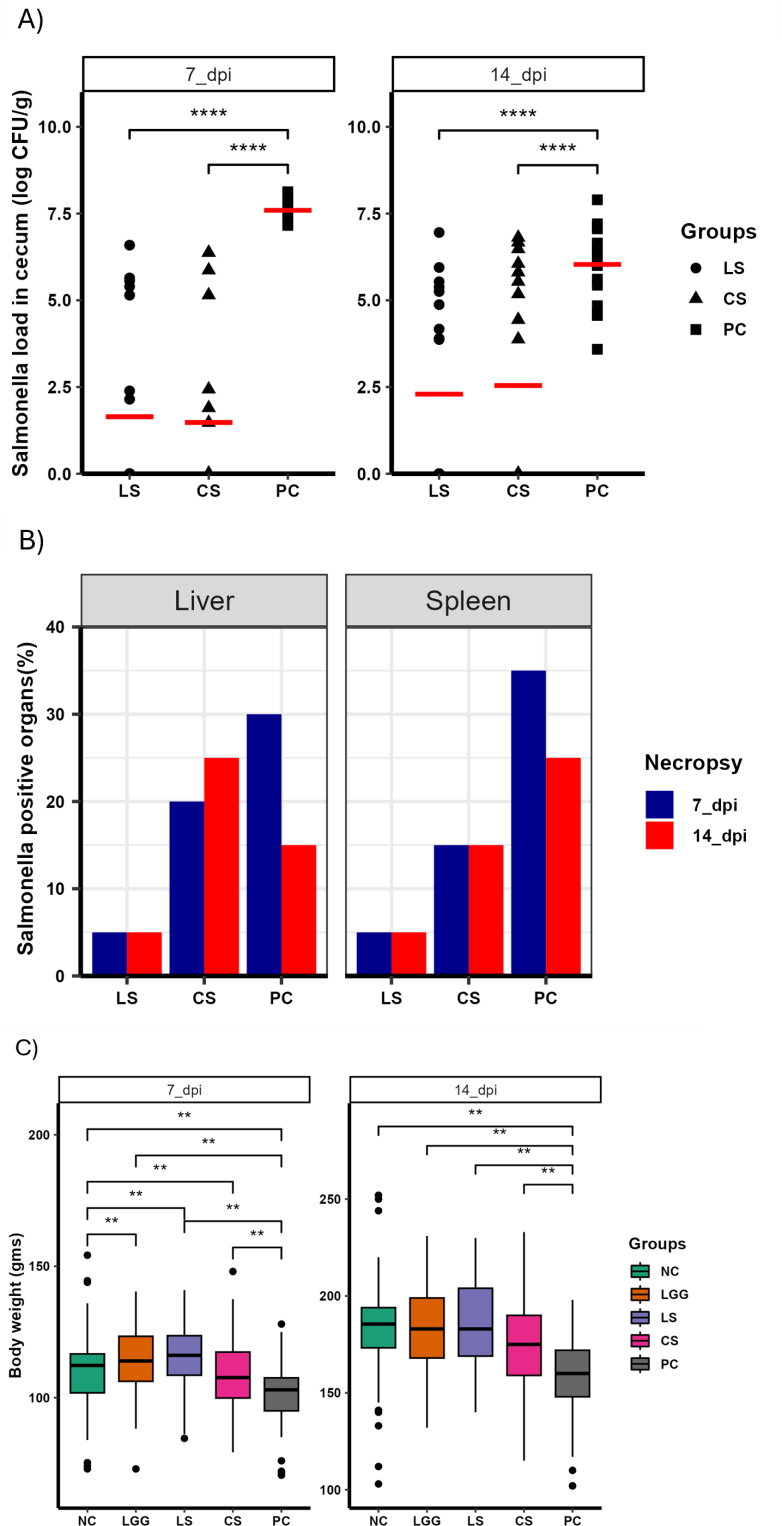
Efficacy of LGG supplemented in drinking water of ST infected chickens raised on the floor. Birds were treated with LGG for 13 days and orally challenged with 10^4^ CFU Nal^r^ ST on day 7. The five groups included were NC, LGG, LS, CS (infected with *Salmonella* and treated with commercial probiotics), and PC. Two necropsies were performed on 7 dpi (first necropsy) and 14 dpi (second necropsy). (A) Log CFU of ST colonization in cecum, (B) percent positive for ST in liver and spleen, and (C) body weight of chickens on 7 and 14 dpi, *N* = 20 birds/group.

We further evaluated the effectiveness of LGG in chickens raised on the built-up litter floor to simulate field conditions. Chickens were continuously treated with either LGG or commercial probiotics starting from day 1 to day 13 for 13 days and infected with nalidixic acid-resistant (Nal^r^) *Salmonella* on day 7. The protective effect of LGG against *Salmonella* infection in chickens was determined in cecum, liver, and spleen. On 7 dpi, the load of *Salmonella* in the cecum of PC, LS, and CS (treated with commercial probiotic and infected with *Salmonella*) groups was 7.59, 1.64, and 1.48 logs, respectively (Fig. 6A). Likewise, on 14 dpi, the load of *Salmonella* in the cecum of PC, LS, and CS was 6.03, 2.29, and 2.54 logs, respectively (Fig. 6A). The load of *Salmonella* in the liver and spleen was determined qualitatively after enrichment. On 7 dpi, 30%, 5%, and 20% of the liver were positive for *Salmonella* in the PC, LS, and CS groups, respectively (Fig. 6B). Similarly, on 14 dpi, 15%, 5%, and 25% of the liver were positive for *Salmonella* in the PC, LS, and CS groups, respectively (Fig. 6B). On 7 dpi, 35%, 5%, and 15% of the spleen were positive for *Salmonella* in the PC, LS, and CS groups, respectively (Fig. 6B). Similarly, on 14 dpi, 25%, 5%, and 15% of the spleen were positive for *Salmonella* in the PC, LS, and CS, respectively (Fig. 6B). In contrast to the previous chicken experiments, there was a significant difference in the body weight of chickens between the groups on both 7 and 14 dpi (Fig. 6C). The average body weight of the PC group was lowest compared to all other four groups (Fig. 6C). The difference in body weight observed between different groups might be due to the large number of birds used in this trial.

### LGG increased the cecal bacterial diversity and evenness reduced by *Salmonella* Typhimurium challenge on 14 dpi

**Fig 7:**
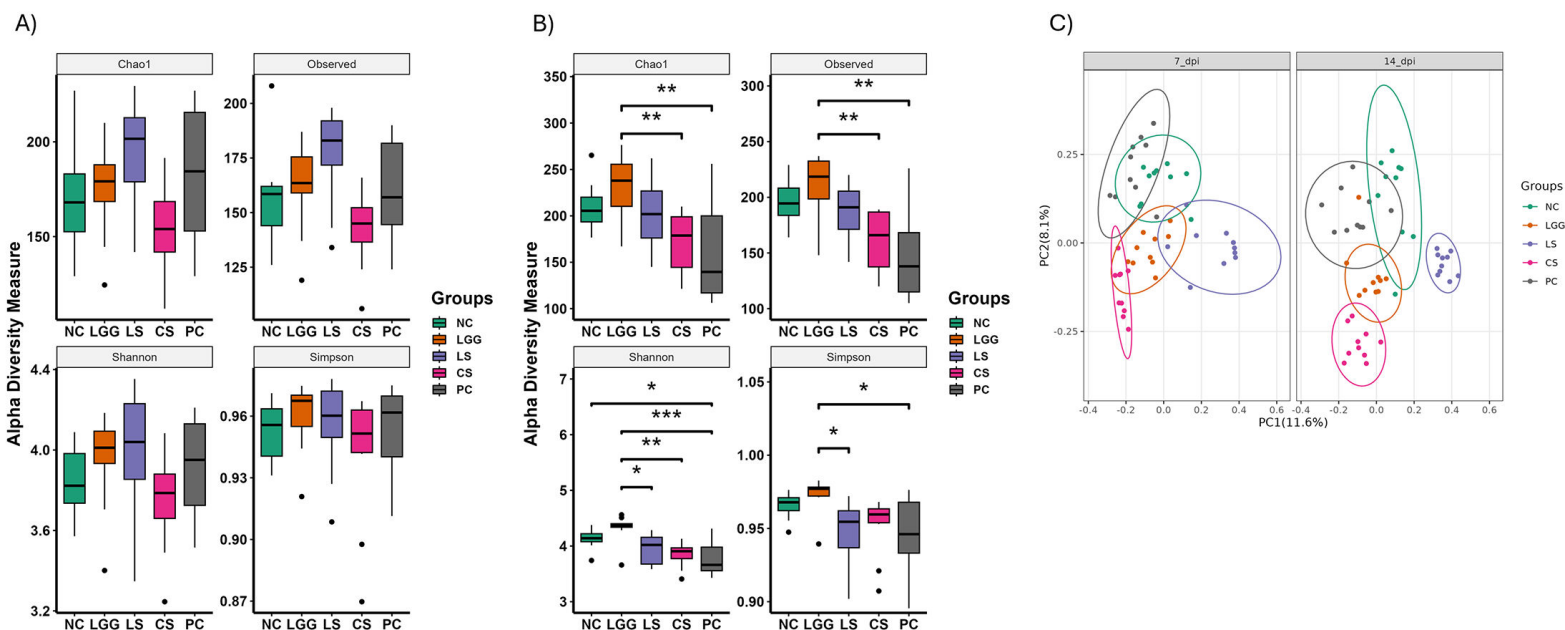
Alpha diversity and beta diversity in cecal microbiota of different groups from chicken trial 3. Influence of LGG in the alpha diversity metrics Chao1, Observed, Shannon and Simpson on 7 dpi (A) and 14 dpi (B). (C) Principal component analysis using Bray-Curtis method of beta diversity on day 7 and 14 dpi.

We hypothesized that one of the mechanisms by which LGG protects chickens against *Salmonella* is via modulation of the gut microbiota. Indeed, a balanced gut microbiota can resist pathogen colonization (28). Therefore, 16s rRNA sequencing was done to assess the changes induced by LGG supplementation in alpha diversity, beta diversity, and relative abundance of cecal microbiota in chickens from the floor trial (chicken trial 3). Alpha diversity, which describes species richness and abundance, was compared between the treatment groups using species richness (Chao1 estimate), diversity measures (Shannon and Simpson indexes), and observed amplicon sequence variants (ASVs). Our results demonstrated that on 7 dpi, although the NC, LGG, and LS groups had greater alpha diversity compared to the PC group, none of the alpha diversity metrics showed significant differences between the treatment groups (Fig. 7A). However, on 14 dpi, there was a significant difference between Chao1, Observed, Shannon and Simpson indices (Fig. 7B). The supplementation of LGG significantly increased the Chao1, Observed, and Shannon index compared to the CS group and PC group (Fig. 7B). Likewise, the Shannon index was significantly higher in the NC group compared to the PC group. There was a non-significant difference in the Choa1 and observed between LS vs any other group (Fig. 7B). However, the LS group had significantly lower Shannon and Simpson indices than the LGG group (Fig. 7B).

To understand the overall variation in microbiome communities between groups, beta diversity was computed using the Bray-Curtis method, and the output was visualized in principal-coordinate analysis (PCoA) faceted by necropsies. The ellipse was used to group the samples of different treatments. Each dot in the PCoA plot represents a single chicken. Our results showed the clear clustering of different groups on both 7 and 14 dpi, indicating that each treatment selected specific species within the group (Fig. 7C). To determine whether the treatment groups were significantly different from each other at two different time points (7 and 14 dpi), permutational multivariate analysis of variance (PERMANOVA) followed by pairwise comparison was applied. Results revealed significant differences among almost all treatment groups except CS vs PC and LGG vs NC on 7 dpi and LS vs NC on 14 dpi (Fig. 7C). The clear differences in microbiota composition among different treatment groups suggest that administration of LGG to *Salmonella*-infected chickens significantly altered the composition and diversity of the cecal microbial communities.

### LGG supplementation modulated the cecal bacteria taxonomical composition

**Fig 8:**
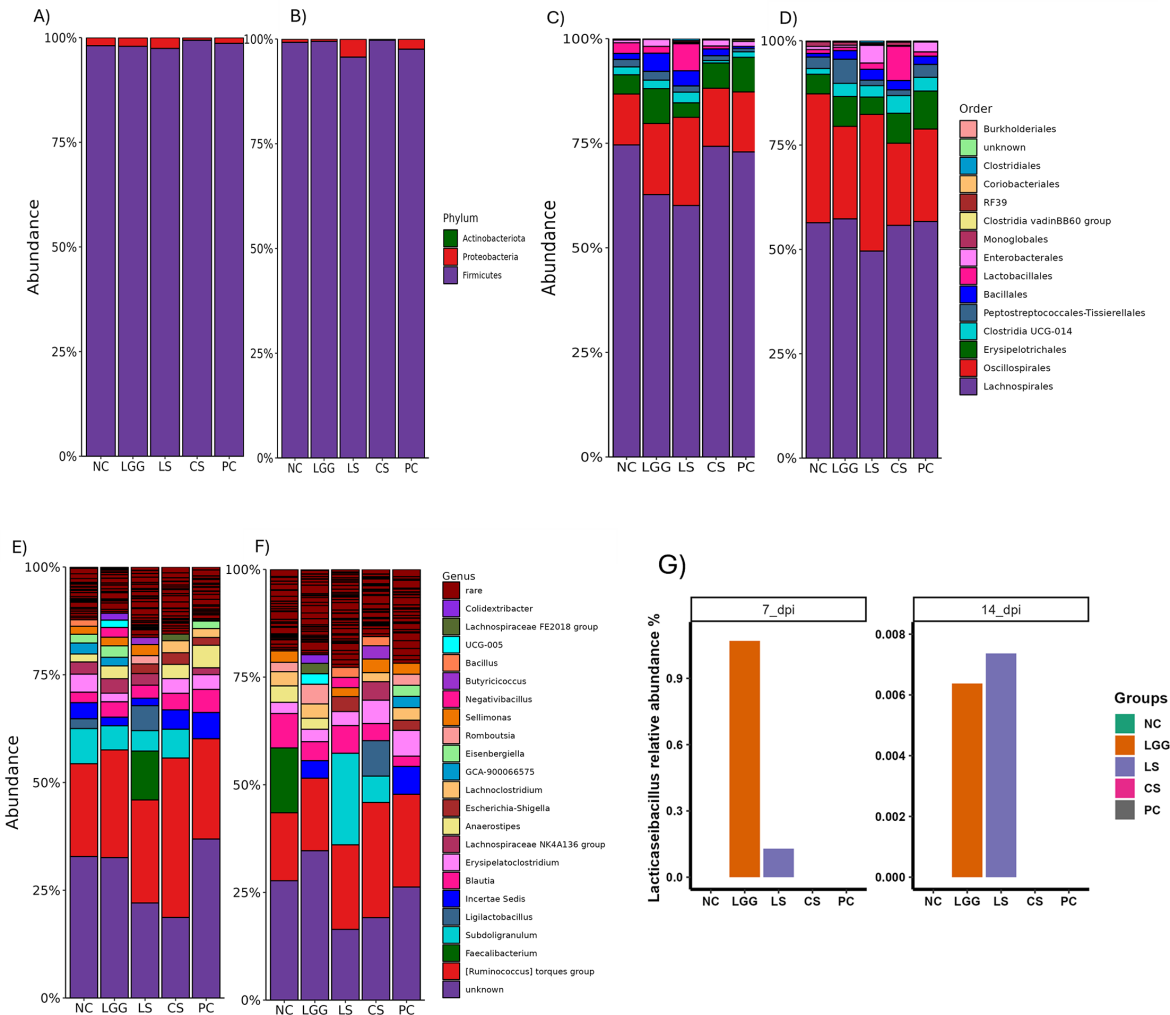
Relative abundance of microbes at two different time points and three different levels: at phylum level on 7 dpi (A) and 14 dpi (B), at order level on 7 dpi (C) and 14 dpi (D), at genus level on 7 dpi (E) and 14 dpi (F). Bar graph showing the relative abundance of *Lacticasebacillus* in different groups (G).

At the phylum level, three major phyla detected were Firmicutes, Proteobacteria, and Actinobacteria on 7 and 14 dpi, which are dominant phyla observed in the cecum of chickens (Fig. 8A and B). Among them, Firmicutes showed the highest abundance (>97%) followed by Proteobacteria and Actinobacteria (Fig. 8A and B). There was no significant difference in the relative abundance of Proteobacteria in the NC vs PC groups on 7 and 14 dpi (Fig. 8A and B). However, the relative abundance of Proteobacteria decreased significantly (*P* < 0.05) in the CS group compared to LGG, LS, and PC groups on both 7 and 14 dpi (Fig. 8A and B).

At the order level, 13 and 14 orders were detected on 7 and 14 dpi, respectively (Fig. 8C and D) (Table S1A and B). The relative abundance of Oscillospirales was significantly higher in the LS group compared to the PC on both 7 and 14 dpi. At the genus level, we detected 66 and 64 unique genera on 7 and 14 dpi, respectively (Fig. 8E and F) (Table S1C and C). Surprisingly, the number of genera detected was lower on day 14, despite the expected increase in microbiota complexity with bird age (35). Our results demonstrated a decreased abundance of *Blautia*, *Shuttleworthia*, *Intestinimonas*, and *Subdoligranulum* in all other groups compared to the PC group on 7 and 14 dpi. The increased abundance of *Butyricicoccus*, *Erysipelatoclostridium*, and *Flavonifractor* was observed in four treatment groups compared to the PC group on 7 and 14 dpi (Fig. 8E and F). Additionally, relative abundance of *Bacillus* increased in the LGG and LS groups compared to the CS and PC groups on 7 and 14 dpi. The relative abundance of *Escherichia-Shigella* was higher in the LS compared to the PC group, whereas it was lowest in the CS group compared to all other groups on 7 and 14 dpi. Although detected at less than 0.01%, genus *Salmonella* was detected in the PC group on 7 dpi but not in other groups. The genus *Lacticaseibacillus* was detected in LGG and LS groups on both 7 and 14 dpi (Fig. 8G). The presence of *Lacticaseibacillus* in the LGG and LS groups was further confirmed by LGG-specific PCR, providing validity to cecal microbiota data.

### LGG administration improved the intestinal histological integrity of chickens

**Fig 9:**
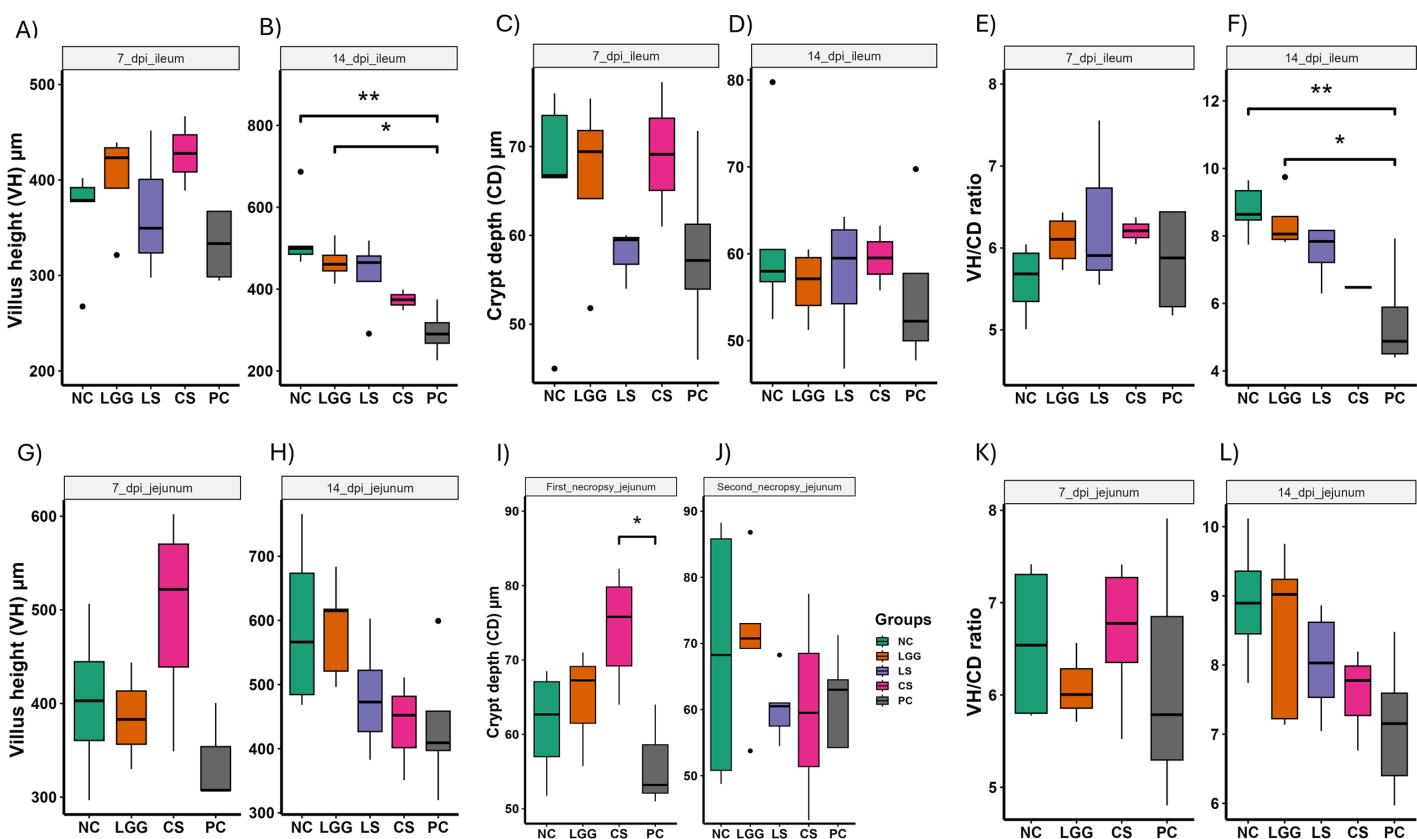
VH and crypt depth (CD) of Ileum (A–F) and Jejunum (G–L). *Salmonella* infection reduced the VH and CD of the ileum, whereas inclusion of LGG significantly increased the VH.

The effects of the supplementation of the LGG and commercial probiotics on the histomorphology of ileum and jejunum are shown in Fig. 9. On 7 dpi, a numerically higher but not statistically significant increase in villus height (VH) of ileum was observed in NC, LGG, LS, and CS groups compared to the PC group (Fig. 9A). On 14 dpi, however, significant differences in the VH were observed between NC vs PC and LGG vs PC groups (Fig. 9B).

A numerically higher but not statistically significant increase in crypt depth (CD) of ileum was observed in NC, LGG, and CS groups on 7 dpi compared to the PC group (Fig. 9C). Likewise, no significant difference in the CD was observed between any groups on 14 dpi (Fig. 9D). The ileal VH:CD ratios appeared proportional to the absolute lengths of ileal VH and CD, and significant differences were observed between NC vs PC and LGG vs PC groups on 14 dpi (Fig. 9E and F). Notably, there were also evident trends toward higher ileal VH and VH:CD ratios in the LS group on 14 dpi compared to the PC group. Therefore, the inclusion of LGG in the drinking water of chickens with or without *Salmonella* infection might have increased or preserved the ileal VH, potentially improving intestinal integrity or nutrient absorption from the gut.

For the jejunum, there was no significant difference in VH of the PC group on 7 and 14 dpi compared to any other groups (Fig. 9G and H). However, the VH of the CS group was numerically higher than any other group on 7 dpi. The CD of CS vs PC group showed a significant difference on 7 dpi, whereas no significant difference in CD was observed in any groups on 14 dpi compared to the PC group (Fig. 9I and J). There was no significant difference in jejunal VH:CD ratios of NC, LGG, or CS groups on 7 and 14 dpi compared to the PC group (Fig. 9K and L). Similar to the ileal VH:CD ratios on 14 dpi, jejunum VH:CD on 14 dpi was numerically higher compared to the PC; however, it was not significant.

### Supplementation of LGG did not induce significant changes in the expression of cytokines and chemokines in the cecal tonsils of chickens

The quantitative real-time polymerase chain reaction (qRT-PCR) was performed on samples from chicken trial 3 to assess the changes in the relative gene expression of several pro-inflammatory cytokines (IFN-g, IL-1b, IL-6, IL-17a, IL-17f), pro-inflammatory and anti-inflammatory cytokine (IL-10), and pro-inflammatory chemokines (ChCXCLI1 and ChCXCLI2) between different groups (36). No significant difference in relative expression of pro-inflammatory cytokines and chemokines was observed on 7 dpi (Fig. S1A). However, the expression of IL-10 was significantly higher in the CS group compared to the PC group on 7 dpi (Fig. S1A). Similarly, the expression level of pro-inflammatory cytokines (IL-1b) was numerically higher in the PC group compared to all other groups at 7 dpi. In contrast, IL-1b was numerically lower in the PC group compared to all other groups on 14 dpi (Fig. S1B).

### Cell-free supernatant of *L. rhamnosus* GG and *B. lactis* Bb12 contain lactic acid and multiple small peptides

**Fig 10:**
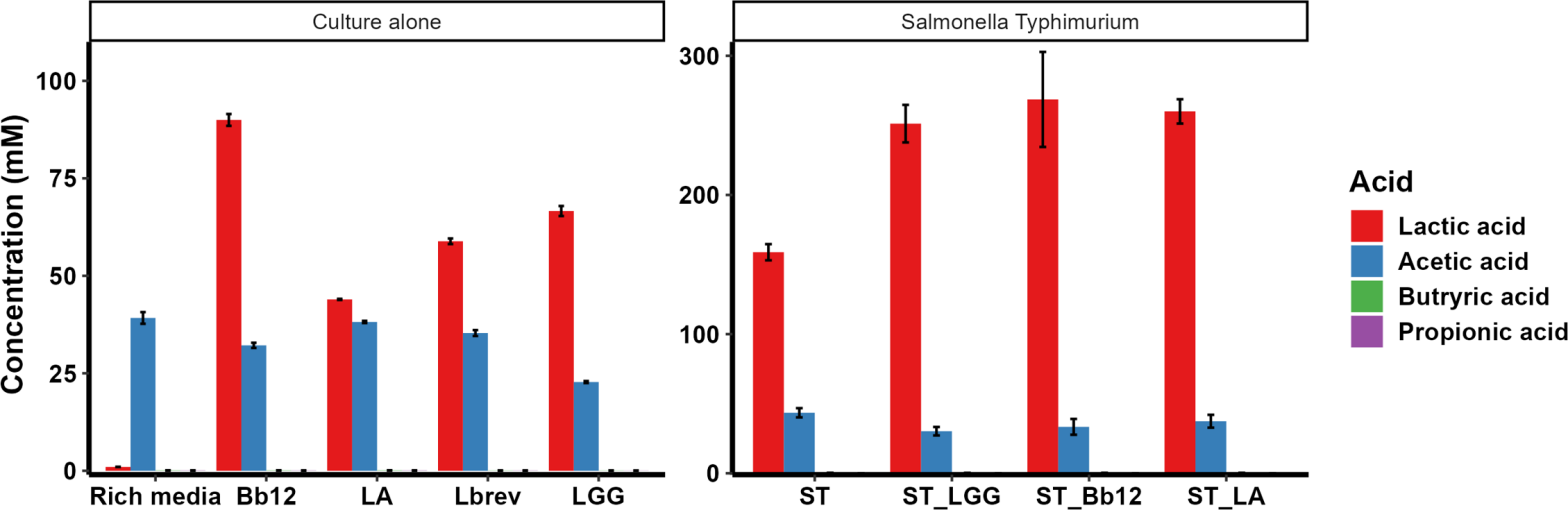
LC-MS/MS profiling of different organic acids in the CFS of probiotic cultured alone and co-cultured with ST. As control, MRS media alone was used as control for monoculture study, and *Salmonella* grown in co-culture media as co-culture study.

The organic acids in CFS of LGG, Bb12, LA*,* and Lbrev probiotics were quantified to investigate the contributing factors of *Salmonella* inhibition. Lbrev and LA were included in the analyses to determine whether these two strains differed in their composition from the effective probiotics LGG and BB12. Liquid chromatography-with tandem mass spectrometry (LC-MS/MS) and isotope-labeled chemical derivatization method were used to identify and quantify the organic acids produced by the probiotics. To quantify the concentration, standard curves were generated for each organic acid (lactic acid: y = 0.0006x − 0.2761, R^2^ = 0.9865, acetic acid: y = 0.0006x − 0.3419, R^2^ = 0.9807, propionic acid: y = 0.00012x − 0.577, R^2^ = 0.9839, and butyric acid: y = 0.0017x − 0.6008, R^2^ = 0.9855). Lactic acid was the major organic acid in the CFS of LA (44 mM), LGG (67 mM), Bb12 (90 mM), and Lbrev (59 mM) (Fig. 10). When the probiotics (LA, LGG, and BB12) were co-cultured with ST, lactic acid still predominated (ST_LA 260.03 mM, ST_LGG 251.24 mM, ST_Bb12 268.62 mM). Acetic acid was the next most abundant organic acid (ST_Bb12 33.36 mM, ST_LA 37.39 mM, ST_LGG 30.24 mM) (Fig. 10).

Furthermore, CFSs were eluted using HyperSep Hypercarb SPE cartridge and analyzed by LC-MS/MS to identify the bioactive molecules in CFS of LA, LGG, Bb12, and Lbrev. A total of 152 peptides and 57 peptides were identified using ion-trap-based collision-induced dissociation (CID) and higher energy collision dissociation (HCD) settings, respectively (Table S2A and B). There were 33 peptides common between the two settings with molecular weight less than 3 KDa (Table S2C) and were found in LGG and Bb12 consistent with their strong anti-*Salmonella* activity.

### Novel peptides identified in CFS of probiotics inhibit ST

**Fig 11:**
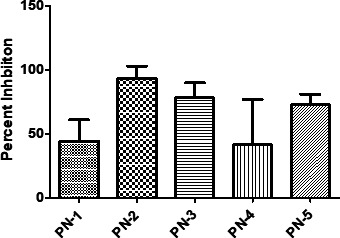
Antimicrobial activity of LGG/Bb12-derived novel peptides against ST. ST (10^5^ CFU/mL) was incubated with PN-1, PN-2, PN-3, PN-4, and PN-5 peptides at 12 mM, and optical density (OD_600_) was recorded using Tecan, an automated microplate reader, every 30 min. ST inhibition by PN-1, PN-2, PN-3, PN-4, and PN-5 was computed as inhibition percentage.

Five peptides were selected for synthesis from the 33 peptides (Table S2C) based on their charge, hydrophobicity, and abundance in LGG and Bb12 to assess the anti-*Salmonella* activity. Abundance in LGG and Bb12 was used as a criterion to select the peptides because of their efficacy against *Salmonella* observed in our previous experiments (Fig. 1 to 3). The selected five peptides were (PN-1: FSAVALSAVALSKPGHVNA, PN-2: AESSDTNLVNAKAA, PN-3: VQAAQAGDTKPIEV, PN-4: AFDNTDTSLDSTFKSA, and PN-5: VTDTSGKAGTTKISNV) commercially synthesized and tested against ST at 12 mM using the 96 well plate (Table S2C). Our results showed that PN-2 showed the greatest inhibition (86%), followed by PN-3 (82%), PN-5 (78%), PN-4 (67%), and PN-1 (38%) at 12 mM as provided in Fig. 11. Molecular weight and retention times of PN-2 (A), PN-3 (B), and PN-5 (C) that showed the highest inhibition are shown in Fig. S2 and S3.

### LGG also inhibited other food safety-relevant *Salmonella* serotypes

The efficacy of LGG was evaluated against different serotypes of *Salmonella* (*S*. Anatum, *S*. Albany, *S*. Brenderup, *S*. Javiana, *S*. Heidelberg, *S*. Muenchen, *S*. Newport, and *S*. Saintpaul) in an agar well diffusion assay using the whole culture of LGG. Our results demonstrated that LGG inhibited diverse *Salmonella* serotypes (Table 4). The largest inhibition was observed with *S*. Muenchen (17 ± 0.50 mm).

**TABLE 4:** Anti-*Salmonella* activity of LGG against *Salmonella* serovars in agar-well diffusion assay^*a*^

*Salmonella* serotype	24 h zone of inhibition (mm ± SD)
*S*. Anatum	14 ± 0.0
*S*. Albany	14 ± 0.0
*S*. Brenderup	13 ± 0.50
*S*. Javiana	14 ± 0.00
*S*. Heidelberg	14 ± 0.50
*S*. Muenchen	17 ± 0.50
*S. N*ewport	14 ± 0.00
*S*. Saintpaul	13 ± 0.00

^*a*^ Serotype of *Salmonella* (~5 × 10^7^ CFU/mL) was inoculated on agar plates and 100 µL of LGG was aseptically punctured on the plate. The inhibition zone was measured after 24 h.

## DISCUSSION

Despite the implementation of several *Salmonella* control programs, the transmission of foodborne salmonellosis remains a significant concern globally in the food animal production systems and humans (5, 37). Poultry serves as the primary reservoir of *Salmonella* transmission to humans. The emergence of antimicrobial resistance genes in *Salmonella*, lack of cross-protection by vaccines against diverse serotypes, higher production cost, and the rapid degradation of the bacteriophages underscores the urgent need for novel approaches to control *Salmonella* infection in poultry (5, 38). Probiotics, which are live beneficial microorganisms when administered in an optimum amount, have received tremendous attention in recent years as potential alternatives due to their several beneficial properties. Studies have reported that probiotics can reduce the load of *Salmonella* in poultry**,** either directly through the production of the bactericidal molecules such as organic acids and bacteriocins or indirectly via the competitive exclusion of pathogens, modulation of gut microbiota, regulation of immune gene expression, and improvement of gut integrity (39–41). Thus, to determine the most effective probiotics with anti-*Salmonella* property, we evaluated the efficacy of five different probiotics against *Salmonella in vitro*. Among them, two probiotics demonstrating the most potent inhibitory activity across various *in vitro* conditions were further tested *in vivo*.

To evaluate the efficacy of probiotics against *Salmonella in vitro*, agar well diffusion assay was performed using both whole culture and CFS. The whole culture and CFS of LGG and Bb12 were able to inhibit the growth of *Salmonella* in an agar well diffusion assay (Tables 1 and 3). This finding was consistent with other studies indicating that probiotics can inhibit *Salmonella* in agar well diffusion assays (42, 43). Moreover, these studies demonstrated that LGG inhibited the growth of multiple Gram-negative bacteria including *S*. Typhimurium, *E. coli*, *Shigella sonnei*, *Staphylococcus aureus*, and *Clostridium difficile* (42, 43). Similar to the results observed in our study (Tables 1 and 3), *Lactobacillus paracasei* subsp. *paracasei* M5-L and *L. rhamnosus* J10-L exhibited thermostability, retaining their inhibitory effects against *Shigella*, *Salmonella*, and *E. coli* even after exposure to elevated temperatures in agar well diffusion assay (42). Probiotics which are used as a feed ingredient in poultry should be heat stable because the feed pelleting usually occurs at very high temperature. This suggests that the inclusion of LGG and Bb12 in the poultry feed will probably retain their efficacy. Furthermore, our results demonstrated antagonistic effects of probiotics against ST and SE after 24 h in co-culture assay (Fig. 1). This corroborates similar inhibitory effects observed when LGG was co-cultured with *Salmonella* (44). Likewise, Abdel-Daim et al. observed reduced number of *S*. Typhi after co-culturing with *Lactobacillus* for 24 h (45).

Recently, the *in vivo* administration of *Lactobacillus* to control *Salmonella* infection has drawn significant attention (46–48). Studies have found that administration of *Lactobacillus* reduced the load of *Salmonella* in poultry (47), pigs (48), and mice (46). In our study, oral administration of LGG significantly reduced the colonization of ST in the cecum by ~1.9 logs on 10 dpi. Our result is consistent with other studies examining the role of lactic acid bacteria (LAB) against *Salmonella* infection in poultry (39, 49, 50). The oral administration of 10^6^ and 10^8^ CFU of the *Lactobacillus* spp. in the neonatal broiler chicks reduced the load of *Salmonella* Enteritidis (51). Similarly, *Lactobacillus* administered 1-h post-infection reduced *Salmonella* Heidelberg levels in chickens and turkey poults (52). Furthermore, the oral administration of LGG significantly reduced the load of Avian pathogenic *E. coli* in chickens (53). In contrast to cecum data, the percentage of the internal organs that became positive for *Salmonella* after oral supplementation of LGG was inconsistent (Fig. 4 to 5). In the LS group, few liver samples tested positive for *Salmonella* compared to the PC group (Fig. 5; chicken trial 2), while the same percentage of liver samples were positive for *Salmonella* compared to the PC group (Fig. 4; chicken trial 1). In the LS group, few spleen samples tested positive for *Salmonella* compared to the PC group (Fig. 4; chicken trial 1), while the same percentage of spleen samples were positive for *Salmonella* compared to the PC group (Fig. 5; chicken trial 2). Similar to our findings, a study evaluating the efficacy of Bacillus-based probiotics against the *Salmonella*-infected chickens demonstrated inconsistent percentages of birds positive for *Salmonella* in internal tissues (39). In poultry, probiotics are combined to potentiate the benefits of each probiotic against the pathogen infection (54). Combination of three LAB probiotics as a batch culture (1:1:1 probiotic mix) significantly reduced *S*. Typhimurium cecal colonization by 1 log in turkey poults (50). Our results showed that despite nearly a 2-log reduction in cecal *Salmonella* load in the group supplemented with LGG alone, combining LGG and Bb12 did not enhance their efficacy (Fig. 4A). The efficacy of LGG was further assessed by administering it in the drinking water of chickens raised in cages and on the floor. Our results showed that administration of probiotics in drinking water significantly reduced the load of *Salmonella* in the cecum under both conditions. Since *Salmonella* can persist in the gut of the poultry and are excreted in the feces for extended periods, LGG administration can reduce the load of *Salmonella* in the gut, reducing the risk of carcass contamination and thus horizontal transmission to humans.

The presence of healthy gut microbiota is crucial to maintain the host health. Gut dysbiosis during *Salmonella* infection in chickens is associated with the lower abundance of the beneficial microorganisms belonging to genera *Blautia*, *Shuttleworthia*, *Subdoligranulum,* and higher abundance of *Butyricicoccus*, *Erysipelatoclostridium*, and *Flavonifractor* (19). The colonization of the gut by the beneficial microbiota reduces sites for the colonization by pathogenic bacteria (55). In addition, gut microbiota has been reported as a producer of vitamins, short-chain fatty acids, organic acids, and antimicrobial peptides, which directly or indirectly inhibits the growth of pathogens (55). However, the disruption of the microbial community makes the host more susceptible to infection (41). Therefore, cecal microbial composition and relative abundance were analyzed after administration of LGG to investigate the role of cecal microbiota in protecting chickens against *Salmonella* infection. Our results demonstrated that the LGG group had significantly higher richness and evenness compared to PC group on 14 dpi (Fig. 7B). Interestingly, at the order level, the relative abundance of *Oscillospirales* was significantly higher in the LS (21% to 14.3%) and (32.7% to 22.1%) group compared to the PC group at 7 and 14 dpi, respectively (Table S1A and B). *Oscillospirales* has been described as a producer of butyric acid and catalase in broilers (56). Several studies have reported that the supplementation of butyric acid reduces the colonization and shedding of *Salmonella* in chickens (56). Wang et al. showed the positive correlation of *Oscillospirales* with catalase production and VH in chickens (57). Catalase is an antioxidant that scavenges the free radicals generated in the body (58). Thus, we speculate that LGG inclusion improves the redox homeostasis in the intestine and improves the gut integrity resulting in enhanced performance of birds.

At the genus level, LGG showed increased abundance of *Bacillus* compared to PC on 7 dpi (1.4% to 0%) and 14 dpi (1.92% to 1.82%) (Table S1C). Similarly, the relative abundance of *Bacillus* was higher in the LS group compared to PC on 7 dpi (5.79% to 0%) and 14 dpi (2.33% to 1.83%) (Table S1D). A recent study showed that the administration of the *Bacillus*-based probiotic in the chicken feed reduces the *Salmonella* load in the feces and internal organs. In addition, *Bacillus* supplementation improved the diversity and abundance of gut microbiota in chickens (59). *Bacillus*-based probiotics were used to restore the gut microbiota of chickens infected with *Salmonella* (19). Therefore, we suggest that the reduction in *Salmonella* load in the LS group compared to the PC group could be due to the higher abundance of *Bacillus. Lacticaseibacillus* was only detected in the LGG (1.06) and LS group (0.12), but not detected in any other groups on 7 dpi (Fig. 8G). A similar trend was observed on 14 dpi, with *Lacticaseibacillus* detected only in the LGG (0.006375112%) and LS group (0.007365723%) (Fig. 8G). This finding was further validated by PCR. Detection of *Lacticaseibacillus* only in the LGG and LS groups even after 8 dpi in both cecal microbiota data and PCR suggests that LGG can colonize in the chicken gut. We also observed an increase in the relative abundance of *Blautia* in LGG on 7 dpi (5.6% to 0.9%) and 14 dpi (4.4% to 2.4%) compared to PC. Similarly, higher abundance of *Blautia* was observed in LS on 7 dpi (4.7% to 0.9%) and 14 dpi (6.4% to 2.4%) compared to PC. *Blautia* are responsible for the production of short-chain fatty acids, anti-inflammatory effect, and scavenge free hydrogen (60, 61).

*Salmonella* is known to induce the mucosal damage of the intestine (51), especially the most distal segment of the small intestine, ileum, so the intact intestine is a barrier for the invasion of the *Salmonella*. Once *Salmonella* is ingested, it damages intestinal integrity, penetrates intestinal epithelial cells, and translocates to internal organs either through the bloodstream or lymphatic system (62). Enhancing intestinal integrity may serve as a strategy to mitigate the pathogenicity of *Salmonella* and its systemic spread to internal organs. The length of VH and CD and VH:CD ratios are some of the parameters to assess the intestinal integrity of poultry (63). Probiotic administration in chickens has been shown to increase intestinal VH and VH:CD ratios, suggesting a protective role against intestinal damage caused by *Salmonella*. In our study, the ileum exhibited a significantly higher VH and VH:CD ratio in the LGG group on 14 dpi compared to the PC group, as well as numerically higher VH and VH:CD ratio in the LS group on 14 dpi compared to the PC group (Fig. 9). However, the jejunum showed no significant difference in VH, CD, and VH:CD ratio between treatment groups on both 7 and 14 dpi (Fig. 9). Previous studies have demonstrated a longer VH in groups challenged with *Salmonella* and treated with probiotics, compared to only the *Salmonella*-infected group, consistent with our findings (41, 47). The longer VH is indicative of the improved intestinal integrity and higher absorption of nutrients (41, 64). Therefore, we suggest that the increase in the body weight of the chicken observed in the LGG and LS groups, along with the reduced load of *Salmonella* in the LS group, might be attributed to the improved intestinal integrity.

One of the mechanisms for the protection mediated by probiotics administration in chickens is via the modulation of immunogenic genes (65). *Salmonella* infection in chickens triggers the expression of several proinflammatory and anti-inflammatory cytokines in the cecal tonsil (65, 66). IFN-g is a proinflammatory cytokine produced by T helper cells and natural killer cells that clears *Salmonella* in chickens (66). The expression of IFN-g is usually upregulated in chickens infected with *Salmonella*, while supplementation of probiotics reduces its expression (65). However, we did not observe any significant changes in IFN-g level in the cecal tonsil of chickens, between the probiotics-treated and non-treated groups on both 7 and 14 dpi (Fig. S1A and B). The absence of detecting significant changes in the IFN-g expression levels could be due to the lower infecting dose (10^4^ CFU) of *Salmonella* used in our study. Most of the studies showing the significant differences in the expression of these cytokines have challenged birds with higher doses of *Salmonella*. For instance, Adhikari et al. observed a significant difference in the IFN-g levels in chickens infected with 10^8^ of *Salmonella* Typhimurium (67); Hsu et al. (68) and Peng et al. (28) challenged birds with 10^10^ CFU of *Salmonella* Typhimurium. To detect the marked difference in the expression of IFN-g and various immune genes in the *Salmonella*-infected vs other groups, further investigations are needed by challenging birds with a higher dose of *Salmonella*.

The anti-*Salmonella* activity of probiotics could be attributed to several factors such as the acidic pH, production of organic acids (e.g., lactic acid, acetic acid, and propionic acid) and anti-microbial peptides (24, 69, 70). Although previous studies have reported that acidic pH is associated with antimicrobial activity of probiotics (24, 70), our data indicate that pH alone was unlikely to account for *Salmonella* inhibition. In the co-culture study, even though Lbrev co-cultured with ST and SE reached an acidic environment after 24 h (Table 2), there was no inhibition in the growth of the *Salmonella* by Lbrev compared to LGG and Bb12 (Fig. 1).

The acidic pH detected in the co-culture of probiotics and *Salmonella* is due to lactic acid and acetic acid produced in both LGG and Bb12 grown with ST (Fig. 10). This is consistent with the De Keersmaecker et al. (44), which reported high levels of lactic acid in the supernatant of LGG. Organic acids, in adequate concentrations, possess antimicrobial activity against pathogenic bacteria; undissociated forms of organic acid can enter the bacterial cell (71). Once inside the cell, organic acid dissociates, generating the proton that lowers the pH of the cytoplasm and kills the pathogenic bacteria (71). The anti-*Salmonella* property observed in our study was not sensitive to proteinase K, aligning with the findings of other studies (72, 73). Furthermore, the production of AMPs is another critical factor that increases the anti-*Salmonella* activity of probiotics. Our results demonstrated that three peptides isolated from the supernatant of probiotics at 12 mM inhibited ST growth more than 70% after 12 h. Similar to our result, several studies have shown the efficacy of AMPs against *Salmonella*-infected mice (74) and chickens (75). AMPs can serve as a promising alternative to conventional antibiotics due to their broad-spectrum activity and lower risk of resistance development (76). The small molecular weight of AMPs enables them to traverse the outer membrane and circumvent other defense mechanisms of Gram-negative bacteria (77). Based on our data, we suggest that the *in vitro* inhibition of *Salmonella* could be due to synergy between the production of organic acids and antimicrobial peptides.

In summary, LGG and Bb12 exhibited superior probiotic properties in *in vitro* conditions such as inhibitory activity, thermostability, and resistance to proteinase K. The *in vivo* results suggest that supplementation of LGG, either orally or in the drinking water, reduces the load of *Salmonella* in the cecum and the internal organs of the chickens. Moreover, the observed anti-*Salmonella* activity of LGG in chickens could be potentially due to the improvement of intestinal integrity, production of organic acids and antimicrobial peptides, and modulation of gut microbiota, as suggested by our *in vivo* and *in vitro* findings. Furthermore, the supplementation of LGG improved the body weight of the chickens. These findings indicate that LGG (53) supplementation can improve the growth and prevent *Salmonella* infection in poultry and thereby enhance food safety and public health.

## MATERIALS AND METHODS

### Bacterial strains and growth conditions

Bacterial strains and their growth conditions used in this study are provided in Table S3. *Salmonella* Typhimurium LT2 (ST-human) (John Gunn, OSU, Columbus) and *Salmonella* Enteritidis (SE-poultry) (laboratory collection) were used to study the antagonistic ability of probiotics. ST and SE were grown in Luria Bertani (LB) broth at 37°C for 18–24 h with shaking at 180 rpm. Nal^r^ ST and SE were generated through spontaneous mutation by plating ST and SE on LB agar containing 100 µg/mL of nalidixic acid (78). *L. acidophilus* NCFM (LA; David Francis, SDSU), *L. rhamnosus* GG (LGG; ATCC 53703), *L. brevis* (Lbrev) (David Francis, SDSU), and *B. animalis* subsp. *lactis* (Bb12; Christian Hansen, Ltd., Hørsholm, Denmark) were cultured using MRS (de Man, Rogosa, and Sharpe) media under anaerobic conditions; MRS was supplemented with 0.05% cysteine hydrochloride for Bb12. Anaerobic conditions for the probiotic strains were maintained using the GasPak EZ Anaerobe Container System Sachets (BD Diagnostics, NJ, USA) (79). LA, LGG, Lbrev, and Bb12 were grown at 37°C for 18–24 h under stationary condition without shaking (80). *Escherichia coli* Nissle 1917 (EcN) (Dr. Ulrich Sonnenborn, Ardeypharm GmbH, Herdecke, Germany) was grown in LB broth under aerobic conditions.

### Agar well diffusion assay using whole culture of probiotics

The agar well diffusion method, as described previously (81), was adapted and used to determine the anti-*Salmonella* ability of commensal bacteria against SE and ST. One hundred microliters of each overnight grown probiotic bacteria (LA, LGG, Bb12, Lbrev, and EcN) adjusted to optical density (OD) of 1 were placed in the wells (aseptically punctured) of ST or SE (~5 × 10^7^ CFU/mL) inoculated agar plates. The inhibition zones (mm) were measured at 12 and 24 h after plates were incubated at 37°C under aerobic conditions. The whole cultures of probiotic bacteria that showed inhibition were then heated to 121°C (autoclaved) and cooled to 4°C for 30 min to determine temperature tolerance of their antibacterial effect. Media-only controls were used to ensure the MRS media did not cause the inhibition.

### Efficacy of probiotics in co-culture assay

To determine the antagonistic effect of Bb12, LA, Lbrev, and LGG against SE and ST, a co-culture assay was conducted. Each probiotic (100 µL of 10^8^ CFU/mL) was individually co-cultured with (100 µL of ~5 × 10^7^ CFU/mL) ST or SE in 7 mL of co-culture (MRS–LB, 1:1 ratio) media and incubated at 37°C anaerobically for 24 h as described previously (44). For enumeration, the co-cultures were plated on LB plates containing nalidixic acid at 0, 6, 12, and 24 h. The experiment was repeated once. The pH of each co-culture was measured at each time point. ST or SE inoculated MRS–LB, ST, or SE grown in LB alone, and media alone were used as controls.

### Trans-well migration assay

The effective probiotics (LGG and Bb12) in the previous experiments were used to test if the antimicrobial activity is due to the bacteria cells or secreted products by trans-well assay (54). A 750 µL of 16–18 h grown LGG and Bb12 cultures were placed above the filter in a 0.22 µm Ultrafree-MC micro-centrifuge filter tube (Millipore Sigma, Burlington, MA, U.S.A). Simultaneously, 750 µL of ST or SE culture (~5 × 10^7^ CFU/mL) were placed below the filter.750 µL of *Salmonella* was sufficient to allow contact with the tube containing the filter. The filter tube was incubated at 37°C under anaerobic conditions with shaking at 50 rpm. A positive control with 750 µL of corresponding *Salmonella* was placed above the filter and used for growth comparison. ST and SE were enumerated on LB plates at 12 and 24 h post-incubation. The experiment was repeated two times.

### Inhibitory activity of CFS of probiotics and effect of temperature and proteinase K on the activity of CFS

The CFS of LGG and Bb12 were assessed for the inhibitory property against *Salmonella* by agar well diffusion assay, as described above. For the preparation of CFS, probiotics were grown overnight, adjusted to OD_600_ 1.0, and sub-cultured in new media for 24 h at 37°C. The CFS were prepared through centrifugation at 10,000 × *g* for 10 min at 4°C and then filtered through a 0.22 µm filter. CFS of LGG and Bb12 were either heated to 121°C (autoclaved), cooled to 4°C (refrigerated), or treated with proteinase K (1 mg/mL, 37°C for 3 h) as described (42, 82) to characterize the stability of antimicrobial activity to temperature and proteinase K. The treated CFS were then subjected to agar well diffusion assay as described above with the controls. A 3 kDa Amicon Ultra centrifugal filter was used to fractionate LGG and Bb12 CFS product(s) of mol wt <3 kDa. The filtrate was used to confirm inhibition of ST in agar well diffusion (54). Proteinase K treatment and subsequent inhibition assay were repeated for SE.

### Effect of probiotic cell-free supernatant on the invasion of *Salmonella* in polarized HT-29 cells

Polarized human colorectal adenocarcinoma (HT-29; ATCC HTB-38) cells were used to determine the effect of LA, LGG, and Bb12 CFS on the invasion of *Salmonella*. HT-29 cells were maintained at 37°C in a humidified atmosphere with 5% CO_2_ in complete DMEM (Gibco, Thermo Fisher Scientific, MA, USA) supplemented with 10% fetal bovine serum (FBS, Gibco), 5 mM galactose, 2 mM L-glutamine, 1% penicillin-streptomycin (PS), and 0.1 mM non-essential amino acids (83, 84). After formation of the monolayer, the cells were maintained with no antibiotic-added media for 2 h. The HT-29 cells were then treated for 4 h with 12.5% and 25% CFS obtained from filtering (3 KDa Amicon filter, Millipore Sigma, Burlington, MA) the culture supernatants of 24 h grown probiotic cultures dissolved in MRS. A 25% and lower concentration of CFS were used as they were non-toxic to HT-29 cells as well as non-inhibitory to *Salmonella* (data not shown). Following pre-treatment with CFS, cells were washed with Dulbecco’s Phosphate-Buffered Saline (DPBS), infected with ST and SE (100-multiplicity of infection) and incubated for 3 h. For infection, ST and SE were grown to mid-logarithmic phase (85), pelleted, washed with DPBS, and re-suspended in DMEM adjusting OD_600_ to 0.05 (5 × 10^7^ CFU/mL). To remove extracellular *Salmonella*, infected cells were washed thrice with DMEM and incubated with DMEM containing 150 µg/mL gentamicin for 1 h (83). Untreated *Salmonella* infected cells were used as controls. The HT-29 cells were washed twice with DPBS, lysed with 0.1% of Triton X-100, and invaded *Salmonella* was enumerated on LB plates supplemented with nalidixic acid (as described earlier) to evaluate the probiotics’ ability to inhibit ST/SE’s invasion of the cells.

### Effect of LGG and Bb12 administered orally against ST infected chickens (chicken trial 1)

The animal study was approved by The Ohio State University Animal Care and Use Program and performed following the Institutional Animal Care and Use Committee protocol #2010A00000149. Fifty-one-day-old SPF leghorn chickens were obtained from a *Salmonella*-free flock at The Ohio State University and randomly allocated into five different groups with 10 birds in each group and housed in cages; NC (neither infected nor treated), LS (infected with *Salmonella* and treated with LGG), BS (infected with *Salmonella* and treated with Bb12), LB_S (infected with *Salmonella* and treated with a mixture of LGG and Bb12 at the ratio of 1:1), and PC (infected with *Salmonella*). Birds were provided with feed and water *ad libitum*. Chickens were treated with 200 µL of LGG, Bb12, or a mixture of LGG and Bb12 in PBS (10^8^ CFU/chicken) daily using oral gavage from day 1 until day 16 of age. On day 7, chickens were orally infected with approximately 10^4^ Nal^r^ ST. On day 17, chickens were euthanized, weights were recorded, and the cecum, liver, and spleen were aseptically collected to determine the probiotics' ability to reduce the load of ST. The cecum was suspended in PBS, homogenized, 10-fold serially diluted, and plated on Xylose-Lysine-Tergitol 4 (XLT4) agar plates supplemented with 50 µg/mL nalidixic acid and incubated for 18 h at 37°C. The liver and spleen were suspended in PBS, and the undiluted homogenized suspension was plated directly and incubated for 18 h at 37°C. Additionally, 1 mL of the undiluted homogenized liver and spleen suspensions were enriched in 9 mL of tetrathionate broth for 18 h at 37°C. After incubation, liver and spleen were plated on XLT4 agar supplemented with nalidixic acid to determine the birds positive for ST in each tissue.

### Effect of LGG administered in the drinking water of ST-infected chickens raised in cages (chicken trial 2)

This chicken trial was conducted in a similar way as described before. A total of 60-day-old SPF layer chickens (*n* = 15/group) obtained from The Ohio State University were divided into four groups and were provided with feed and water *ad libitum*. The groups were NC (neither infected nor treated), LGG (not infected but treated with LGG), LS (infected with *Salmonella* and treated with LGG), and PC (infected with *Salmonella*). Each group had 15 birds housed in the cages. LGG (10^8^ CFU/mL) was administered from day 1 to day 13 of age continuously in the drinking water, changed daily. The volume of drinking water needed was calculated and adjusted based on the standard requirements of chickens (86). Birds were orally challenged with 10^4^ CFU Nal^r^ ST on day 7. On day 15, chickens were euthanized, and the cecum, liver, and spleen were aseptically collected to determine the anti-*Salmonella* activity of probiotics administered in drinking water. The tissues were processed using the aforementioned procedure.

### Effect of LGG administered in the drinking water of ST-infected chickens raised on the floor (chicken trial 3)

This chicken trial was conducted in a similar way as described above. A total of 400 one-day-old SPF leghorn chickens were randomly allocated in five different groups with 80 birds in each group. The groups included were NC (neither infected nor treated with LGG), LGG (not infected but treated with LGG), LS (infected with *Salmonella* and treated with LGG), CS (infected with *Salmonella* and treated with commercial probiotics), and PC (infected with *Salmonella*). Gut pro (# BVS-N-514425, Best Veterinary Solutions, Ellsworth, IA, USA) was used as a commercial probiotic. Gut pro was selected as a probiotic control because it contained *Lactobacillus* in its formulation. The birds were administered 10^8^ CFU/mL of LGG and commercial probiotic by supplementing in the drinking water from day 1 to day 13. On day 7, birds were challenged with 10^4^ CFU of Nal^r^ ST orally. Birds were necropsied on days 7 and 14, post-infection. Cecal tonsils, cecum, liver, spleen, ileum, and jejunum were collected in sterile condition. One cecum was flash-frozen immediately in liquid nitrogen and frozen at −80°C until used for the microbiota analysis, while another cecum, liver, and spleen were processed following the above protocol. The body weight and the feed consumption were recorded weekly. However, feed consumption and feed-to-gain ratio could not be determined due to the large amount of feed wastage.

### Genomic DNA extraction for sequencing of V4/V5 region of 16S rRNA gene

For microbiota analysis, the whole cecum from trial 3 was homogenized and DNA was extracted using 2 g of homogenized tissue using a DNA Purification Kit following manufacturer’s instructions (PureLink Microbiome; Thermo Fisher Scientific, MA, USA). The concentration and quality of DNA were measured using the NanoDrop 2000 C Spectrophotometer (Thermo Fisher Scientific, MA, USA). A portion of 5 ng/µL of diluted DNA samples were submitted for sequencing at the Molecular and Cellular Imaging Center (MCIC) (https://mcic.osu.edu/genomics/illumina-sequencing). PCR amplification of the V4/V5 regions of rRNA was performed using a Ready-Mix PCR Kit (IFU KAPA HiFi HotStart, Roche Sequencing and Life Science, MA, USA). The forward and the reverse primers for the amplification were GAGTGCCAGCMGCCGCGGTAA and ACGGACTACHVGGGTWTCTAAT. The purification of the PCR products was done using Agincourt AMPure XP beads (Beckman Coulter, CA, USA). Nextera XT DNA library preparation kit (Illumina) was used to prepare the library and sequenced in Illumina MiSeq platform generating paired end reads of 300 bp.

### Microbiota analysis

All code used for the metabarcoding analyses can be found in our GitHub repository (https://github.com/jelmerp/salmonella_metabc). The quality of raw FASTQ files was assessed using fastqc (version 0.11.8) (87) and multiqc (version 1.11) (88), and primers were removed using cutadapt (version 3.4) (89). The count table with each ASV was generated using the R/Bioconductor package DADA2 (version 1.16) (90). These steps consist of filtering and trimming to remove the poor quality reads and poor quality bases using the (filterAndTrim() function), condensing the reads that consist of the same sequence using the (derepFastq() function), learning error rates using the (learnErrors() function), inferring ASVs using the (dada() function), merging forward and reverse reads using the (mergePairs() function), constructing the ASV table using the (makeSequenceTable() function), removing the chimera created during the PCR amplification using the (removeBimeraDenovo() function), assigning taxonomy to the ASVs using the (assignTaxonomy() and addSpecies() functions) using the Silva database (version 138.1, available at https://zenodo.org/record/4587955).

For the downstream analysis, the count table, taxonomy table, phylogenetic tree, and a metadata table were stored as a single R object using the phyloseq (version 1.38.0) (91) package in R. Alpha diversity was computed using the plot_richness() function in R. Beta diversity was computed using the Bray-Curtis distance in R. The difference in beta diversity between the treatments was computed by PERMANOVA. DESeq2 version 1.35.0 (92) was used to compute the differential abundance analysis at the genus, order, and class level.

### LGG specific PCR

Our cecal microbiota analysis detected LGG in the LGG and LS chicken groups on both 7 and 14 dpi (chicken trial 3). To validate the result, LGG-specific PCR was conducted following the previously described protocol (93). DNA extracted for the 16S rRNA sequencing was used as a template for the PCR. The reaction mixture of 25 µL consists of 12.5 µL of Taq KeenGreen 2X Master Mix (IBI Scientific, USA), 8.5 µL nuclease-free water, 1 µL of each LGG specific forward (CAATCTGAATGAACAGTTGTC) and reverse (TATCTTGACCAAACTTGACG) primers, and 2 µL of DNA. The amplification program consists of 94°C for 2 min, 35 cycles of 94°C for 30 s, 56°C for 30 s and 72°C for 30 s, followed by final extension at 72°C for 10 min. PCR amplicon was visualized in 1% agarose gel containing 0.5 µg/mL ethidium bromide. PCR mixture without DNA and DNA from LGG non-treated groups on both 7 and 14 dpi were used as controls.

### Gut histomorphometry analysis

During necropsy of chicken trial 3, two ileal and jejunal segments (approximately 2 cm each) were collected from all birds, fixed with 4% paraformaldehyde and stored at room temperature until analyzed (83). The intestinal samples (both ileum and jejunum) were dehydrated with alcohol, cleared with xylene, and embedded in paraffin wax. The paraffin-embedded tissues (three different pieces/ileal or jejunal tissue) were sectioned at 3 µm using a Leica microtome, fixed on slides, and stained using hematoxylin and eosin. The VH and the CD were measured by a pathologist (K. Jung) using the NIH ImageJ program. The VH was measured from the tip of the villus to the villus-crypt junction, and CD was measured from the bottom of the villus to the lamina propria. Then, the VH/CD ratio was calculated.

### Total RNA extraction and real-time quantitative PCR

Changes in gene expression of several genes involved in the immune response in the cecal tonsils were assessed by qRT-PCR. Cecal tonsils from chicken trial 3, NC, LGG, LS, CS, and PC groups (*n* = 10) were stored in RNA later until the extraction of RNA. Total RNA was extracted using TRIzol reagent (Invitrogen, Carlsbad, CA, USA) following the manufacturer’s protocol. The concentration and quality of RNA were measured by nanodrop spectrophotometer (ND-2000, Thermo Fisher Scientific). A 1 µg of RNA was used to synthesize cDNA using the RT^2^ cDNA synthesis kit (Qiagen, MD, USA) following the manufacturer’s protocol. A 25 ng of cDNA was used in each well to amplify the gene sequence. The primer sequences of genes are provided in Table S4 (83)). qRT-PCR was performed on the RealPlex2 master cycler (Eppendorf, CT, USA) using a commercial SYBR Green kit (Applied Biosystems SYBR Green Universal Master Mix). The thermal cycling condition consists of an initial activation phase first at 50°C for 2 min and then at 95°C for 2 min, followed by 40 cycles of 95°C for 15 s for denaturation, 55°C for 15 s for annealing, and 72°C for 1 min for extension. The changes in relative gene expression were normalized using GAPDH as a control gene, and the fold change of each target gene was calculated as 2^−ΔΔCt^.

### Quantification of organic acids in probiotics

As described previously (94), LC-MS/MS was used with an isotope-labeled chemical derivatization method to quantify the organic acids present in LA, LGG, Bb12, and Lbrev. LC-MS/MS analyses were performed at The Mass Spectrometry and Proteomics Facility (https://www.ccic.osu.edu/MSP), The Ohio State University. To prepare CFS of the probiotics, bacteria was grown overnight, adjusted to OD 1 (10^9^ CFU/mL), and 500 µL was sub-cultured in 14.5 mL of fresh media for 24 h in anaerobic condition. LC-MS/MS analysis was conducted on CFS using LC-MS/MS Poroshell 120 SB C18 column with solvent A; H_2_O + 0.1% formic acid and solvent B; MeCN + 0.1% formic acid. Lactic acid, acetic acid, propionic acid, and butyric acid (Sigma Aldrich) were used as standard solutions, and sodium ^13^C-lactic acid was used as the internal standard. This protocol was repeated with CFS of probiotics co-cultured with ST. The CFS of probiotics was prepared by growing LGG and Bb12 anaerobically, centrifuging for 1,000 rpm for 10 min, and resuspending pellets in sterile water with 2% glucose. The culture was incubated for 24 h under anaerobic conditions, centrifuged for 1,000 rpm for 10 min at 4°C and filtered through a 0.22 µm filter (53).

### Identification of bioactive antimicrobial peptides derived from probiotics

LGG and Bb12 supernatants were used to identify derived peptides using LC-MS/MS as described previously (95). The bacteria were grown at 37°C for 24 h under anaerobic conditions, centrifuged (1,000 rpm, 10 min, 25°C), and washed with sterile water. The pellets were then re-suspended in sterile water containing 2% glucose and incubated for 24 h. The probiotic cultures were then centrifuged (1,000 rpm, 10 min, 4°C) and the culture-free supernatants were separated and filtered using a 0.22 µm filter. The supernatants (volume: 1.8 mL) were passed through HyperSep Hypercarb SPE cartridge (50 mg; ThermoFisher Scientific, MA, USA) three times. The cartridge was then washed twice with 150 µL of water to remove salts and then eluted twice (20 µL) using 50% acetonitrile (MeCN) and 0.1% trifluoroacetic acid. The eluted solutions (0.5 µL) were then injected in LC-MS/MS EasySpray C18-Fusion column set at different collision energy (HCD and CID) settings. The LC-MS/MS analysis was conducted at the Mass Spectrometry and Proteomics Facility, The Ohio State University (https://www.ccic.osu.edu/MSP). The two solvents used were solvent A (H_2_O + 0.1% formic acid) and solvent B (Acetonitrile [MeCN] + 0.1% formic acid). Proteome Discoverer 2.2 software (Thermo Fisher Scientific, MA, USA) was used to analyze the data using the UniProt *Lactobacillus* or *Bifidobacterium* database with settings of no modifications and non-specific cleavage.

Five peptides in the CFS of both LGG and Bb12 (FSAVALSAVALSKPGHVNA: PN-1, AESSDTNLVNAKAA: PN-2, VQAAQAGDTKPIEV: PN-3, AFDNTDTSLDSTFKSA: PN-4, and VTDTSGKAGTTKISNV: PN-5) were selected for synthesis based on their charge, hydrophobicity, and abundance in LGG and Bb12. Peptides were commercially synthesized in GenScript (GenScript, Piscataway, NJ, USA) and tested for their inhibitory ability against *Salmonella* in liquid media in a 96-well plate (96, 97). Briefly, peptides dissolved in dimethyl sulfoxide (DMSO) at 12 mM were added to *Salmonella* suspension (10^5^ CFU/mL) in a 96-well plate and incubated at 37°C in a TECAN Sunrise (TECAN, Zurich, Switzerland) absorbance microplate reader with OD_600_ measured every 30 min for 12 h. Sterile media (NC) and DMSO-treated *Salmonella*-infected wells (PC) were used as controls. OD values in the PC wells were used to calculate the growth inhibitory activity of the peptides using (OD_600_ PC − OD_600_ peptide-treated well)/OD_600_ PC × 100%.

### Effect of LGG on multiple *Salmonella* serotypes

Multiple *Salmonella* serotypes (laboratory collection) that are commonly isolated from humans, animals, or produce were used in an agar well diffusion assay to test the efficacy of LGG against them following the above protocol. Serotype of *Salmonella* (~5 × 10^7^ CFU/mL) was spread on agar plates, and 100 uL of LGG was aseptically added to the well on the plate. The inhibition zone was measured after 24 h. The serotypes tested included *S*. Anatum, *S*. Albany, *S*. Brenderup, *S*. Javiana, *S*. Heidelberg, *S*. Muenchen, *S*. Newport, and *S*. Saintpaul.

### Statistical analyses

All the graphs and the statistical analysis were done in R v. 4.3.1 and GraphPad PRISM Version 5. Student two-tailed *t*-test analyses (*P* < 0.05) were conducted for co-culture and cell culture invasion assays. The statistically significant difference of *Salmonella* load in the cecum of chickens was determined using one-way analysis of variance followed by Duncan’s multiple range test. The Wilcoxon test was performed for statistical analysis of alpha diversity and bacterial taxonomic groups (phylum, family, and genus) between the treatments. PERMANOVA was used to calculate significant differences in beta diversity between the treatments. The significant differences were represented as *P < 0.05, **P < 0.01, and ***P < 0.001, throughout the study.

## Data Availability

All data generated or analyzed are included in this published article. The 16S rRNA gene sequencing data were submitted to NCBI under Bioproject PRJNA1127034.

## SUPPLEMENTAL MATERIAL

Supplemental figures iai.00420-24-s0001.pdf Fig. S1 to S3.

Supplemental tables iai.00420-24-s0002.docx Table S1A, 1B, 1C , 1D, S2C, S3 and S4.

Table S2A and S2B iai.00420-24-s0003.xlsx List of small peptides detected in LC/MS.

ASM does not own the copyrights to Supplemental Material that may be linked to, or accessed through, an article. The authors have granted ASM a non-exclusive, world-wide license to publish the Supplemental Material files. Please contact the corresponding author directly for reuse.
