# Supplementary material for: The probiotic Lacticaseibacillus rhamnosus GG supplementation reduces Salmonella load and modulates growth, intestinal morphology, gut microbiota, and immune responses in chickens
Source: Infect Immun. 2025 Apr 2;93(5):e00420-24. doi: 10.1128/iai.00420-24 (PMC12070740; doi:10.1128/iai.00420-24)
Supplement: Supplemental figures — Fig. S1 to S3. [file iai.00420-24-s0001.pdf]

Supplementary Figure 1A

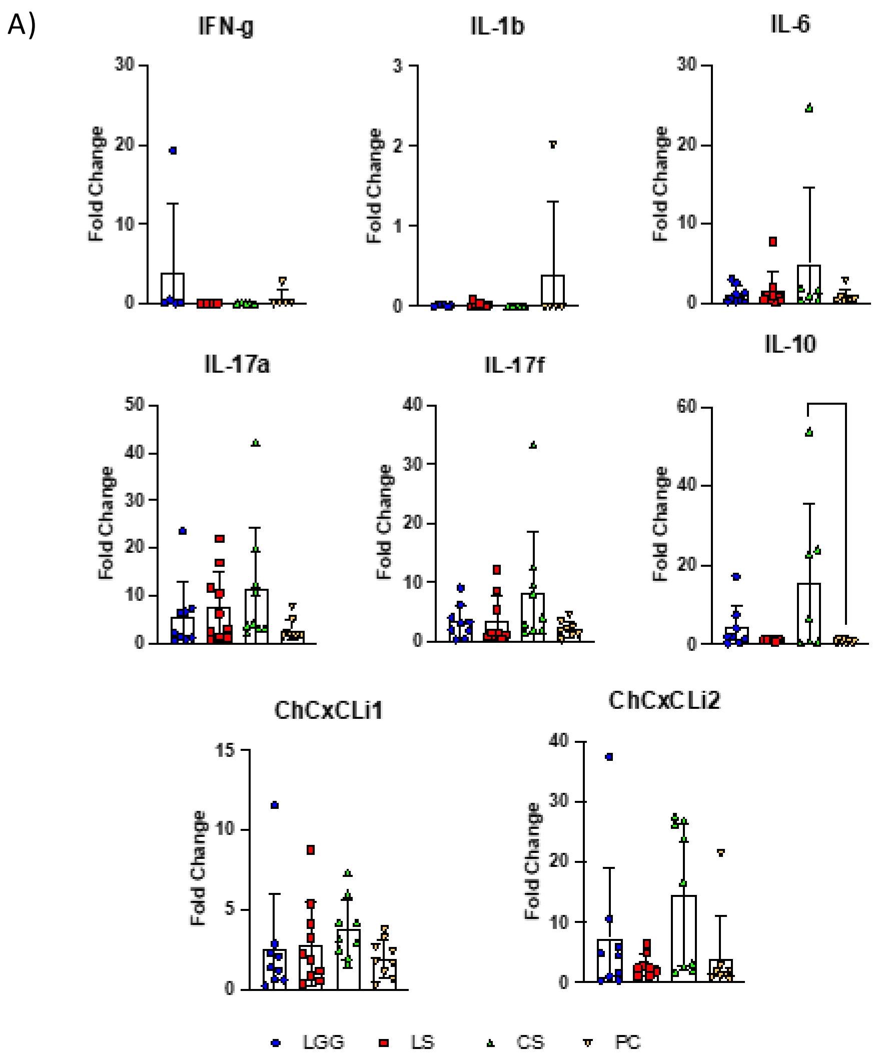

Supplementary Figure 1B

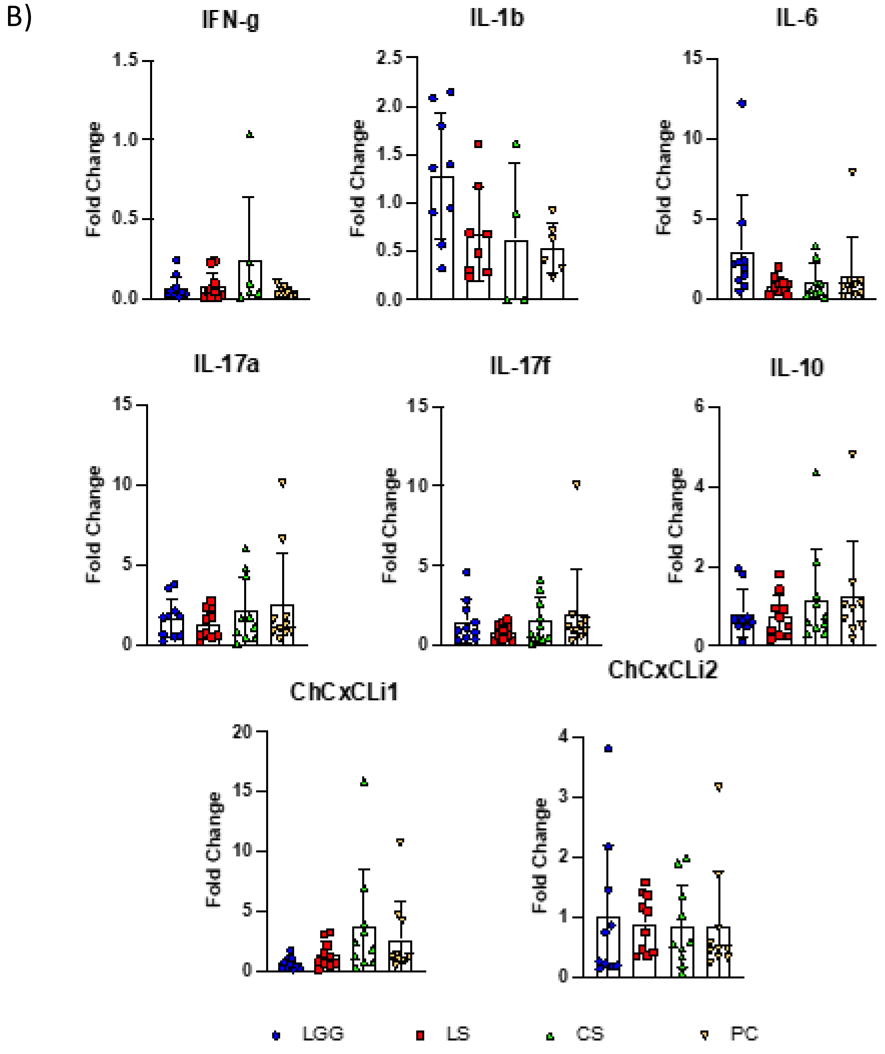

Supplementary Figure 1: Changes in relative expression of the selected chemokine and cytokine immune gene in the cecum tonsils of chickens on 7dpi (A) and 14 dpi (B).

Supplementary Figure 2

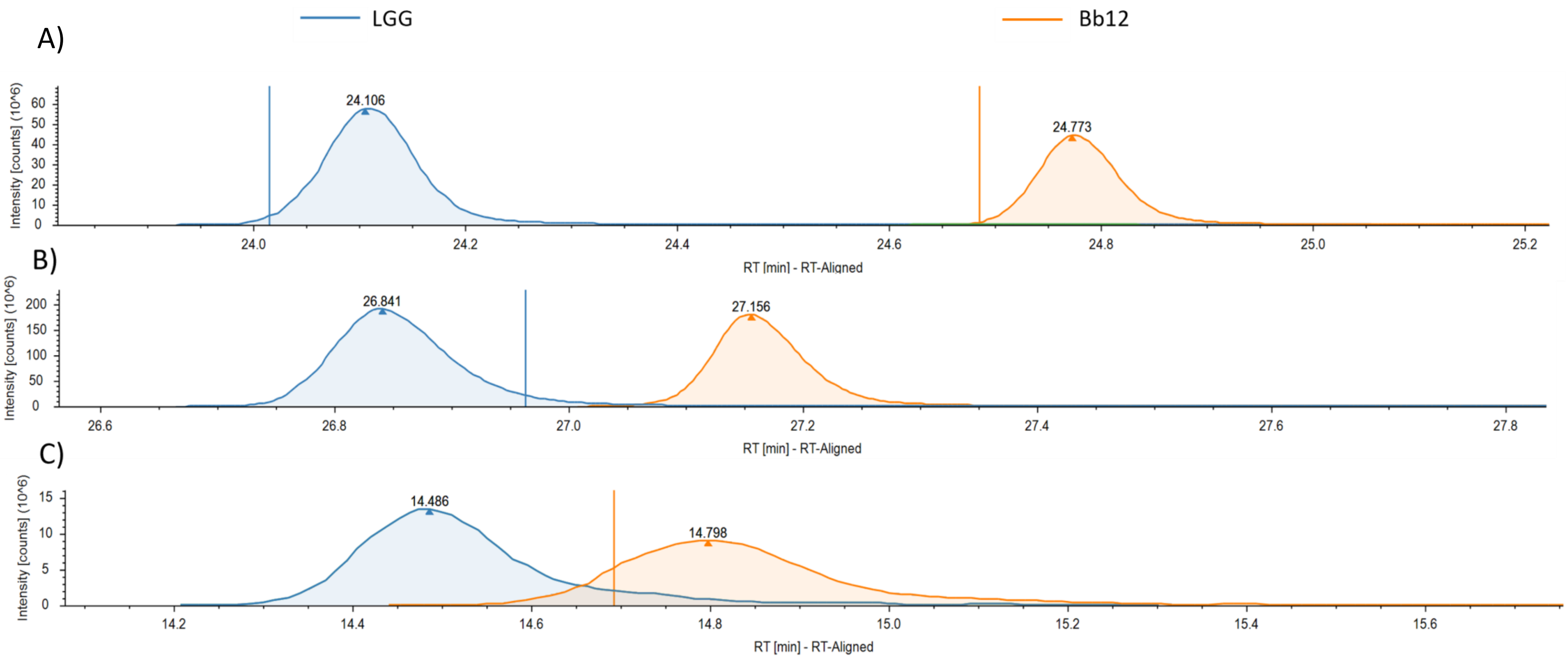

A) PN-2: AESSDTNLVNAKAA B) PN-3: VQAAQAGDTKPIEV C)PN-5: VTDTSGKAGTTKISNV

Supplementary Figure 2: LC-MS peaks and retention times of derived peptides that showed growth inhibitory activity against ST A) PN-2: AESSDTNLVNAKAA B) PN-3: VQAAQAGDTKPIEV C) PN-5: VTDTSGKAGTTKISNV.

Supplementary Figure 3

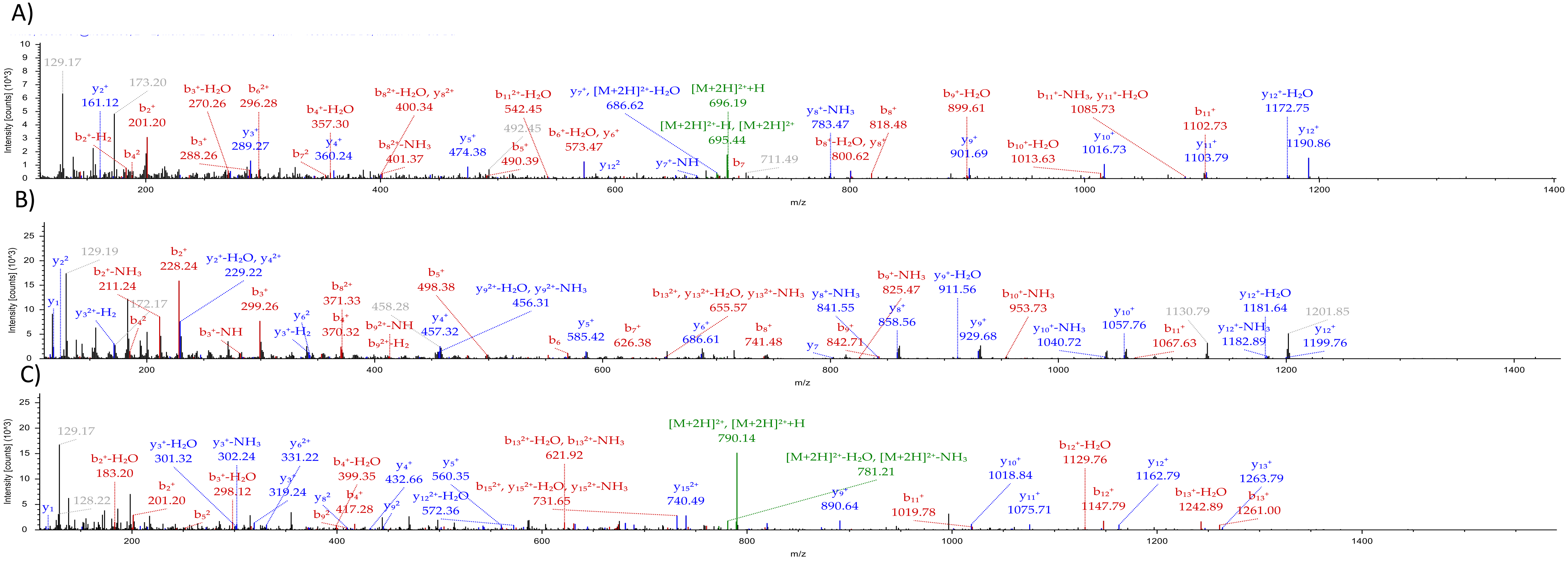

A) PN-2: AESSDTNLVNAKAA B) PN-3: VQAAQAGDTKPIEV C)PN-5:VTDTSGKAGTTKISNV

Supplementary Figure 3: Derived peptides fragmentation peaks used for sequence/ nomenclature. A) PN-2: AESSDTNLVNAKAA B) PN-3: VQAAQAGDTKPIEV C) PN-5: VTDTSGKAGTTKISNV.
