# Supplementary material for: The probiotic Lacticaseibacillus rhamnosus GG supplementation reduces Salmonella load and modulates growth, intestinal morphology, gut microbiota, and immune responses in chickens
Source: Infect Immun. 2025 Apr 2;93(5):e00420-24. doi: 10.1128/iai.00420-24 (PMC12070740; doi:10.1128/iai.00420-24)
Supplement: Supplemental tables — Table S1A, 1B, 1C , 1D, S2C, S3 and S4. [file iai.00420-24-s0002.docx]

***Lacticaseibacillus rhamnosus* GG (LGG) reduces *Salmonella* colonization in chickens**

**Supplementary Table 1:** The relative abundance percentage of order (A : 7dpi and B : 14dpi) and top 20 genus (C : 7dpi and D : 14dpi) identified in the caeca, NC=Negative control, LGG: group treated with LGG, LS: group challenged with *Salmonella* and treated with LGG, CS: group challenged with *Salmonella* and treated with commercial probiotics*,* PC: group challenged with *Salmonella*.

**A)**

| **Order - 7dpi** | **NC** | **LGG** | **LS** | **CS** | **PC** |
| --- | --- | --- | --- | --- | --- |
| Lachnospirales | 74.60 | 62.72 | 60.12 | 74.25 | 72.92 |
| Oscillospirales | 12.16 | 17.00 | 21.07 | 13.86 | 14.33 |
| Erysipelotrichales | 4.60 | 8.33 | 3.46 | 6.04 | 8.26 |
| Clostridia UCG-014 | 1.44 | 4.36 | 3.62 | 1.64 | 0.50 |
| Lactobacillales | 0.65 | 1.67 | 0.23 | 1.45 | 1.20 |
| Peptostreptococcales-Tissierellales | 1.77 | 2.08 | 1.48 | 1.13 | 0.79 |
| Bacillales | 2.56 | 1.64 | 6.47 | 0.71 | 0.00 |
| Enterobacterales | 1.90 | 2.03 | 2.56 | 0.60 | 1.34 |
| Monoglobales | 0.15 | 0.05 | 0.13 | 0.23 | 0.00 |
| Clostridia vadinBB60 group | 0.03 | 0.06 | 0.36 | 0.03 | 0.29 |
| Clostridiales | 0.12 | 0.01 | 0.05 | 0.02 | 0.34 |
| Coriobacteriales | 0.02 | 0.05 | 0.02 | 0.02 | 0.02 |
| RF39 | 0.00 | 0.00 | 0.43 | 0.00 | 0.00 |

**B)**

| **Order - 14dpi** | **NC** | | **LGG** | **LS** | **CS** | **PC** |
| --- | --- | --- | --- | --- | --- | --- |
| Lachnospirales | 56.40 | | 57.33 | 49.61 | 55.76 | 56.68 |
| Oscillospirales | 30.87 | | 22.15 | 32.74 | 19.68 | 22.16 |
| Lactobacillales | 0.92 | | 0.77 | 1.50 | 8.21 | 1.03 |
| Erysipelotrichales | 4.68 | | 7.16 | 4.14 | 7.17 | 9.09 |
| Clostridia UCG-014 | 1.40 | | 3.14 | 2.76 | 4.25 | 3.30 |
| Bacillales | 0.89 | | 2.09 | 2.61 | 2.25 | 1.99 |
| Peptostreptococcales-Tissierellales | 2.73 | | 5.78 | 1.29 | 1.35 | 3.07 |
| Monoglobales | 1.11 | | 0.73 | 0.17 | 0.80 | 0.04 |
| Enterobacterales | 0.76 | | 0.51 | 4.24 | 0.18 | 2.37 |
| RF39 | 0.00 | | 0.08 | 0.30 | 0.14 | 0.00 |
| Coriobacteriales | 0.04 | | 0.04 | 0.07 | 0.09 | 0.04 |
| Clostridia vadinBB60 group | | 0.15 | 0.15 | 0.07 | 0.07 | 0.21 |
| Clostridiales | | 0.05 | 0.03 | 0.49 | 0.02 | 0.01 |
| Burkholderiales | | 0.00 | 0.00 | 0.004 | 0.00 | 0.00 |

**C)**

| **Genus -7dpi** | **NC** | **LGG** | **LS** | **CS** | **PC** |
| --- | --- | --- | --- | --- | --- |
| Unknown | 32.8 | 32.6 | 22.0 | 18.7 | 36.9 |
| *[Ruminococcus] torques group* | 21.5 | 25.0 | 23.9 | 37.0 | 23.3 |
| *Blautia* | 8.2 | 5.6 | 4.7 | 6.7 | 0.9 |
| *Lachnoclostridium* | 4.2 | 2.0 | 1.4 | 3.4 | 3.4 |
| *Sellimonas* | 3.7 | 2.0 | 1.7 | 4.5 | 6.1 |
| *Anaerostipes* | 2.8 | 3.4 | 2.7 | 0.6 | 1.6 |
| *UCG-005* | 2.5 | 2.0 | 0.7 | 0.8 | 0.5 |
| *Erysipelatoclostridium* | 2.4 | 3.6 | 3.0 | 3.8 | 5.4 |
| *Bacillus* | 2.3 | 1.5 | 5.8 | 0.7 | 0.0 |
| *Lachnospiraceae FE2018 group* | 2.0 | 2.7 | 0.7 | 1.4 | 1.7 |
| *Escherichia-Shigella* | 1.9 | 2.0 | 2.6 | 0.6 | 1.3 |
| *Incertae Sedis* | 1.8 | 3.0 | 1.4 | 3.3 | 5.2 |
| *Eisenbergiella* | 1.5 | 0.1 | 0.5 | 0.0 | 0.7 |
| *Shuttleworthia* | 1.3 | 0.0 | 0.1 | 0.0 | 0.0 |
| *Catenibacillus* | 1.2 | 0.0 | 0.0 | 0.0 | 0.0 |
| *Romboutsia* | 1.1 | 1.6 | 1.0 | 0.8 | 0.2 |
| *Flavonifractor* | 0.9 | 0.9 | 0.4 | 1.0 | 1.2 |
| *Oscillibacter* | 0.9 | 0.5 | 0.4 | 0.4 | 0.5 |
| *Butyricicoccus* | 0.6 | 0.5 | 0.6 | 2.8 | 2.1 |
| *Marvinbryantia* | 0.5 | 1.7 | 0.4 | 0.5 | 0.9 |

**D)**

| **Genus - 14dpi** | **NC** | **LGG** | **LS** | **CS** | **PC** |
| --- | --- | --- | --- | --- | --- |
| Unknown | 27.72 | 34.68 | 16.40 | 19.14 | 26.25 |
| *[Ruminococcus] torques group* | 15.77 | 16.83 | 19.65 | 26.73 | 21.52 |
| *Faecalibacterium* | 15.07 | 0.00 | 0.00 | 0.00 | 0.00 |
| *Blautia* | 7.98 | 4.42 | 6.44 | 4.00 | 2.40 |
| *Anaerostipes* | 3.82 | 2.55 | 1.06 | 1.19 | 0.99 |
| *Lachnoclostridium* | 3.29 | 3.34 | 1.74 | 2.12 | 2.91 |
| *Sellimonas* | 2.64 | 1.96 | 2.07 | 3.14 | 2.56 |
| *Erysipelatoclostridium* | 2.60 | 2.88 | 3.27 | 5.42 | 5.99 |
| *Romboutsia* | 2.19 | 4.57 | 0.86 | 1.10 | 2.57 |
| *Incertae Sedis* | 1.79 | 4.08 | 1.13 | 1.92 | 6.48 |
| *[Eubacterium] hallii group* | 1.60 | 0.83 | 0.58 | 1.06 | 1.68 |
| *Eisenbergiella* | 1.48 | 0.89 | 1.20 | 1.19 | 2.58 |
| *Negativibacillus* | 1.39 | 1.11 | 2.37 | 0.00 | 1.68 |
| *Shuttleworthia* | 1.30 | 0.74 | 0.76 | 0.00 | 0.11 |
| *Monoglobus* | 1.11 | 0.73 | 0.17 | 0.80 | 0.04 |
| *Butyricicoccus* | 1.02 | 0.14 | 0.84 | 3.08 | 1.79 |
| *UCG-005* | 0.88 | 2.44 | 0.72 | 0.58 | 1.82 |
| *Bacillus* | 0.78 | 1.93 | 2.33 | 2.13 | 1.83 |
| *Lactobacillus* | 0.69 | 0.00 | 0.00 | 0.00 | 0.00 |
| *Turicibacter* | 0.69 | 0.65 | 0.39 | 0.27 | 0.02 |

| **Supplementary Table 2C:** List of the 33 novel peptides identified in CFS of probiotics in CID and HCD setting | | |
| --- | --- | --- |
| **Sequence** | **Theo. MH + [Da]** | **Accession number** |
| EVKALAEKVLKK | 1355.86 | A0A0R2DJY6 |
| SAVALSAVALSKPGHVNA | 1691.94 | C2JZA7 |
| AVALSAVALSKPGHVNA | 1604.91 | C2JZA7 |
| VALSAVALSKPGHVNA | 1533.87 | C2JZA7 |
| FSAVALSAVALSKPGHVNA* | 1839.01 | C2JZA7 |
| VAGVTLASASTLDKDIKD | 1803.97 | C2JYJ6 |
| LKDVLSSYLSTSSSSSTSK | 1977 | A0A180C684 |
| ALSAVALSKPGHVNA | 1434.81 | C2JZA7 |
| AQNGNTNKIEVDNIVYK | 1919.98 | A0A179YFC2 |
| VAGVTLASASTLDKDVKE | 1803.97 | A0A0R2DLD3 |
| VIVVVAAIGGGLNNKGKSSS | 1870.08 | A0A179YAS6 |
| DEVKALAEKVLKK | 1470.89 | A0A0R2DJY6 |
| GNDTPADSAVKARIV | 1513.8 | K8QAJ2 |
| HDVIQNALNAK | 1222.65 | A0A249DEL5 |
| LSSYLSTSSSSSTSK | 1521.73 | A0A180C684 |
| FSQATNAYFIKGA | 1417.71 | A0A2A5L4H0 |
| AADKSQVKVGVLQL | 1455.85 | C2K1D8 |
| AESSDTNLVNAKAA* | 1390.68 | A0A179YN16 |
| ATLAGVGVSGFAATTVHA | 1629.86 | A0A179XCY0 |
| ALDVDGIIAQLKDA | 1441.79 | A0A0H0YQJ8 |
| VQAAQAGDTKPIEV* | 1426.75 | A0A179YFC2 |
| VNAAQNGNTNKIEVDNIVYK | 2204.13 | A0A179YFC2 |
| VNAAQNGNTNKIEVDNI | 1813.9 | A0A179YFC2 |
| SINRDDYNKAVSDGQDKL | 2037.98 | A0A2A5L4H0 |
| QSQFAQEQSEAAKATQA | 1822.86 | A0A179YJG3 |
| AFDNTDTSLDSTFKSA* | 1719.77 | A0A180C684 |
| AIAAITDTMKKEGLAE | 1661.88 | K0N9I2 |
| DANKIKEQLEEVGATVTLK | 2086.14 | A0A0H0YQJ8 |
| DTSGKAGTTKISNV | 1378.72 | A0A1Z2F669 |
| EVASKTNDIAGDGTTTA | 1650.78 | A0A0R1WMV7 |
| GLALITAVPQVVRA | 1407.87 | A0A179Y5L8 |
| VTDTSGKAGTTKISNV* | 1578.83 | A0A1Z2F669 |
| NKVGPKEYIPELNKSL | 1829.02 | A0A179YFC2 |
| *Peptides selected for synthesis  **Supplementary Table 3:** List of bacteria used in this study, and growth conditions.   \| **Bacterial spp.** \| **Media** \| **Culture conditions** \| **Reference/source** \| \| --- \| --- \| --- \| --- \| \| *Salmonella* Anatum \| LB broth \| 37 °C, aerobic, 12 h, 180 rpm \| Laboratory collection \| \| *Salmonella* Albany \| LB broth \| 37 °C, aerobic, 12 h, 180 rpm \| Laboratory collection \| \| *Salmonella* Brenderup \| LB broth \| 37 °C, aerobic, 12 h, 180 rpm \| Laboratory collection \| \| *Salmonella* Javiana \| LB broth \| 37 °C, aerobic, 12 h, 180 rpm \| Laboratory collection \| \| *Salmonella* Heidelberg \| LB broth \| 37 °C, aerobic, 12 h, 180 rpm \| Laboratory collection \| \| *Salmonella* Muenchen \| LB broth \| 37 °C, aerobic, 12 h, 180 rpm \| Laboratory collection \| \| *Salmonella* Newport \| LB broth \| 37 °C, aerobic, 12 h, 180 rpm \| Laboratory collection \| \| *Salmonella* Saintpaul \| LB broth \| 37 °C, aerobic, 12 h, 180 rpm \| Laboratory collection \| \| *Salmonella* Typhimurium LT2 \| LB broth \| 37 °C, aerobic, 18-24 h, 180 rpm \| John Gunn, OSU, Columbus \| \| *Salmonella* Enteritidis \| LB broth \| 37 °C, aerobic, 18-24 h, 180 rpm \| Laboratory collection \| \| *Levilactobacillus brevis* \| MRS broth \| 37 °C, anaerobic, 1–2 days \| David Francis, SDSU \| \| *Lactobacillus acidophilus* \| MRS broth \| 37 °C, anaerobic, 1–2 days \| David Francis, SDSU \| \| *Lacticaseibacillus rhamnosus GG* \| MRS broth \| 37 °C, anaerobic, 1–2 days \| ATCC, Manassas, VA, USA \| \| *Bifidobacterium lactis Bb12* \| MRS broth + 0.05% cysteine \| 37 °C, anaerobic, 24 h \| Christian Hansen Ltd., Hørsholm, Denmark \| \| *Escherichia coli Nissle 1917* \| LB broth \| 37 °C, aerobic, 10–12 h, 200 rpm \| Dr. Ulrich Sonnenborn, Ardeypharm GmbH, Herdecke, Germany \| | | |

**Supplementary Table 4:** Details of primers used for quantitative real time PCR

| **Target gene** | **Primer sequence (5′-3′)** | **Product size (bp)** |
| --- | --- | --- |
| GAPDH | F: GACGTGCAGCAGGAACACTA | 343 |
|  | R: TCTCCATGGTGGTGA AGACA |  |
| IFN-g | F: TGAGCCAGATTGTTTCGATG | 152 |
|  | R: CTTGGCCAGGTCCATGATA |  |
| IL-1b | F: GGATTCTGAGCACACCACAGT | 272 |
|  | R: TCTGGTTGATGTCGAAGATGTC |  |
| IL-10 | F: GCTGCGCTTCTACACAGATG | 203 |
|  | R: TCCCGTTCTCATCCATCTTC |  |
| IL-6 | F: GCTCGCCGGCTTCGA | 71 |
|  | R: GGTAGGTCTGAAAGGCGAACAG |  |
| IL17-A | F: CATGGGATTACAGGATCGATGA | 68 |
|  | R: GCGGCACTGGGCATCA |  |
| IL17-F | F: TGACCCTGCCTCTAGGATGATC | 78 |
|  | R: GGGTCCTCATCGAGCCTGTA |  |
| ChCXCLi1 | F: CCGATGCCAGTGCATAGAG | 191 |
|  | R: CCTTGTCCAGAATTGCCTTG |  |
| ChCXCLi2 | F: CCTGGTTTCAGCTGCTCTGT | 128 |
|  | R: GCGTCAGCTTCACATCTTGA |  |
